# Supplementary material for: ntsm: an alignment-free, ultra-low-coverage, sequencing technology agnostic, intraspecies sample comparison tool for sample swap detection
Source: Gigascience. 2024 Jun 4;13:giae024. doi: 10.1093/gigascience/giae024 (PMC11148594; doi:10.1093/gigascience/giae024)
Supplement: giae024_GIGA-D-23-00333_Revision_3 [file giae024_giga-d-23-00333_revision_3.pdf]

GigaScience

ntsm: an alignment-free, ultra low coverage, sequencing technology agnostic, intraspecies sample comparison tool for sample swap detection

--Manuscript Draft--

**Manuscript Number:**	GIGA-D-23-00333R3
**Full Title:**	ntsm: an alignment-free, ultra low coverage, sequencing technology agnostic, intraspecies sample comparison tool for sample swap detection
**Article Type:**	Technical Note
**Funding Information:**	National Human Genome Research Institute (U01HG010961) — Dr Heng Li<br>National Human Genome Research Institute (R01HG010040) — Dr Heng Li
**Abstract:**	Background<br><br>Due to human error, sample swapping in large cohort studies with heterogeneous data types (e.g. mix of Oxford Nanopore, Pacific Bioscience, Illumina data, etc.) remains a common issue plaguing large-scale studies. At present, all sample swapping detection methods require costly and unnecessary (e.g. if data is only used for genome assembly) alignment, positional sorting, and indexing of the data in order to compare similarly. As studies include more samples and new sequencing data types, robust quality control tools will become increasingly important.<br><br>Findings<br><br>The similarity between samples can be determined using indexed k-mer sequence variants. To increase statistical power, we use coverage information on variant sites, calculating similarity using a likelihood ratio-based test. Per sample error rate, and coverage bias (i.e. missing sites) can also be estimated with this information, which can be used to determine if a spatially indexed PCA-based pre-screening method can be used, which can greatly speed up analysis by preventing exhaustive all-to-all comparisons.<br><br>Conclusions<br><br>Because this tool processes raw data, is faster than alignment, and can be used on very low coverage data, it can save an immense degree of computational resources in standard QC pipelines. It is robust enough to be used on different sequencing data types, important in studies that leverage the strengths of different sequencing technologies. In addition to its primary use case of sample-swap detection, this method provides other useful information useful in QC, such as error rate and coverage bias, as well as population-level PCA ancestry analysis visualization.
**Corresponding Author:**	Justin Chu<br>Dana-Farber Cancer Institute<br>Boston, MA UNITED STATES
**Corresponding Author Secondary Information:**	
**Corresponding Author's Institution:**	Dana-Farber Cancer Institute
**Corresponding Author's Secondary Institution:**	
**First Author:**	Justin Chu
**First Author Secondary Information:**	
**Order of Authors:**	Justin Chu

Powered by Editorial Manager® and ProduXion Manager® from Aries Systems Corporation

	Jiazhen Rong
	Xiaowen Feng
	Heng Li
**Order of Authors Secondary Information:**	
**Response to Reviewers:**	Editor suggested changes made. Please let me know if there are any issues.
**Additional Information:**	
**Question**	**Response**
Are you submitting this manuscript to a special series or article collection?	No
**Experimental design and statistics**<br><br>Full details of the experimental design and statistical methods used should be given in the Methods section, as detailed in our Minimum Standards Reporting Checklist. Information essential to interpreting the data presented should be made available in the figure legends.<br><br>Have you included all the information requested in your manuscript?	Yes
**Resources**<br><br>A description of all resources used, including antibodies, cell lines, animals and software tools, with enough information to allow them to be uniquely identified, should be included in the Methods section. Authors are strongly encouraged to cite Research Resource Identifiers (RRIDs) for antibodies, model organisms and tools, where possible.<br><br>Have you included the information requested as detailed in our Minimum Standards Reporting Checklist?	Yes
**Availability of data and materials**<br><br>All datasets and code on which the conclusions of the paper rely must be either included in your submission or	Yes

deposited in publicly available repositories (where available and ethically appropriate), referencing such data using a unique identifier in the references and in the "Availability of Data and Materials" section of your manuscript.<br><br>Have you have met the above requirement as detailed in our Minimum Standards Reporting Checklist?	

Powered by Editorial Manager® and ProduXion Manager® from Aries Systems Corporation

Manuscript Click here to access/download;Manuscript;ntsm_20240429.docx

ntsm: an alignment-free, ultra low coverage, sequencing technology agnostic, intraspecies sample comparison tool for sample swap detection

Justin Chu[1,2*], Jiazhen Rong[3], Xiaowen Feng[1,2], Heng Li[1,2*]
ORCID iDs: Justin Chu [0000-0003-0549-4997]; Jiazhen Rong [0000-0002-7884-5973]; Xiaowen Feng [0000-0002-2291-1361]; Heng Li [0000-0003-4874-2874]
Emails: cjustin@ds.dfci.harvard.edu, RongJ@pennmedicine.upenn.edu, xfeng@ds.dfci.harvard.edu, hli@ds.dfci.harvard.edu

Institutional Addresses:

1. Dana-Farber Cancer Institute, 450 Brookline Ave. Boston, MA 02215-5450
2. Harvard Medical School, 25 Shattuck Street, Boston, MA 02115
3. Genomics and Computational Biology Graduate Program, Perelman School of Medicine, University of Pennsylvania

*Corresponding Authors: Justin Chu, Heng Li

Abstract

Background: Due to human error, sample swapping in large cohort studies with heterogeneous data types (e.g. mix of Oxford Nanopore (ONT), Pacific Bioscience, Illumina data, *etc.*) remains a common issue plaguing large-scale studies. At present, all sample swapping detection methods require costly and unnecessary (e.g. if data is only used for genome assembly) alignment, positional sorting, and indexing of the data in order to compare similarly. As studies include more samples and new sequencing data types, robust quality control tools will become increasingly important.

Findings: The similarity between samples can be determined using indexed *k*-mer sequence variants. To increase statistical power, we use coverage information on variant sites, calculating similarity using a likelihood ratio-based test. Per sample error rate, and coverage bias (*i.e.* missing sites) can also be estimated with this information, which can be used to determine if a spatially indexed PCA-based pre-screening method can be used, which can greatly speed up analysis by preventing exhaustive all-to-all comparisons.

Conclusions: Because this tool processes raw data, is faster than alignment, and can be used on very low coverage data, it can save an immense degree of computational resources in standard QC pipelines. It is robust enough to be used on different sequencing data types, important in studies that leverage the strengths of different sequencing technologies. In addition to its primary use case of sample-swap detection, this method also provides information useful in QC, such as error rate and coverage bias, as well as population-level PCA ancestry analysis visualization.

Keywords

Sample mixup, Sample-swap detection, Alignment-free, Sequencing technology agnostic, Quality control, Error rate estimation, PCA-based population analysis, Low sequence coverage analysis

Introduction

Large-scale sequencing studies often have robust error reduction strategies, though none are immune to human error. If sample swaps occur it can be trivial to detect known contaminants using sequence classification tools [1,2], or distance-based analysis such as MASH [3], however sample swaps in intra-species studies can be difficult to detect as the high degree of similarity due to being the same species can overwhelm the signal to distinguish unrelated samples, which can be further confounded by sequencing error or other artifacts caused by batch effects.

For same-species sample swap detection, using prior knowledge of variants with a minor allele frequency (MAF) ideally near 50% within the population can help increase the sensitivity of the analysis to only differences between individuals. For detecting sample swaps between the same species, in particular humans, multiple tools have been developed utilizing variant sites [4–11]. These methods rely on upstream alignment, sorting and indexing of the data, many initially require a variant calling pipeline as well, though at least newer methods such as Somalier [11] do not require a variant calling set working directly on alignments. In addition, even these methods may be overwhelmed when comparing low coverage or heterogeneous data types such as Illumina sequencing [12] and ONT sequencing [13], or specialized library preparation methods upstream of sequencing such as Hi-C [14] or 10x Chromium [15] linked read sequencing data.

Rather than determining sample swaps after the alignment, sorting and indexing of the sequence data, it may be ideal to detect sample swap or other issues at the furthest possible upstream analysis point as to minimize extraneous computational costs. It may be argued that alignment may not incur any additional analysis costs as such things may be part of the downstream analysis anyway, however, studies that do not require alignments exist. For example, the Vertebrate Genome Project (VGP) [16] assembles PacBio High-Fidelity (HiFi) reads and Hi-C short reads in the lack of a known reference genome; the default workflow in the Human Pangenome Reference Consortium (HPRC) [17] also performs assembly without aligning them to the reference human genome. In addition, data specialized for other purposes than variant calling is also difficult to use in these pipelines. In light of these issues, we sought to create a tool that generically detects sample swaps and it is convenient to use upstream of any analyses.

We have created a tool for fast sample swap detection on raw whole genome sequencing data, agnostic of sequencing technology. As it uses only *k*-mer counts in the analysis and lacks the requirement of any alignment and sorting, it is unrivaled in speed compared to traditional alignment based methods, and can function on any kind of sequence data even at very low coverage data as long as the raw data is mostly uniform in coverage. In addition, the *k*-mer count information also provides the extra quality control utility such as error rate estimation and PCA population analysis to determine sample population of origin.

Methods

Algorithm Overview

We developed ntsm focusing on minimizing upstream processing as much as possible. It starts by counting the relevant variant *k*-mers from a sample only keeping information needed to perform the downstream analysis. The counting can be set to terminate early if sufficient *k*-mer coverage is obtained. Once generated the counts can be compared in a pairwise manner using a likelihood-ratio based test. During this, sequence error rate is also estimated using the counts. The number of tests can be reduced by specifying an optional PCA rotation matrix and

normalization matrix adding a prefiltering step on high quality samples. Finally, matching sample pairs are outputted in a tsv file.

Selection of variant sites and *k*-mers for human samples

For tools of this nature to function effectively, it is important to select a robust set of polymorphic sites. In our case we attempt to select sites that primarily have 2 variants within the population, that occur at high (ideally near 50%) population frequencies. For our *k*-mer based method, the sites must also not contain repetitive *k*-mers, as the coverage influences the computed confidence of our statistical test. Finally, in our selection of site for human samples we included other criteria for selection (Figure 1) which, while helpful for various reasons, are less important for our tool to function properly.

The polymorphic sites for human data are initially derived from an intersection of Hapmap3 [18] and Illumina Omni Express v3 [19] SNP chip sites, selected for their practical reliability allowing for possible comparisons of data using only these sites. These candidate sites are then filtered by cross-referencing the dbSNP [20] database to retain sites with a minor allele frequency (MAF) > 0.05. Any sites within 31 bp of each other are also filtered out, as we use *k*-mers that need to be mostly independent of each other in our analysis. Next, we filtered the regions by difficult regions as determined by the Genome in a Bottle Consortium [20,21]. We then keep only (A or T to G or C) variants, as human-error induced DNA strand mix ups are common but less likely when non-complementary base polymorphism are used.

We then process each site pulling out the 19-mers within a 31bp window for each variant and align them to hg38 using bwa aln [22] to find any 19-mers that align multiple times with at most 1 mismatch to ensure we are not using any repetitive 19-mers. Any sites with at least 3 non-repetitive 19-mers within the window are kept, resulting in a final total of 96287 sites. We expect that any similar procedure to create sites for another organism will benefit from a similar filtering step to minimize the effects of repetitive sequences. As applications for human samples are expected to be quite common, we have provided the sequences for these sites with respective identifiers (rsIDs) along with our tool.

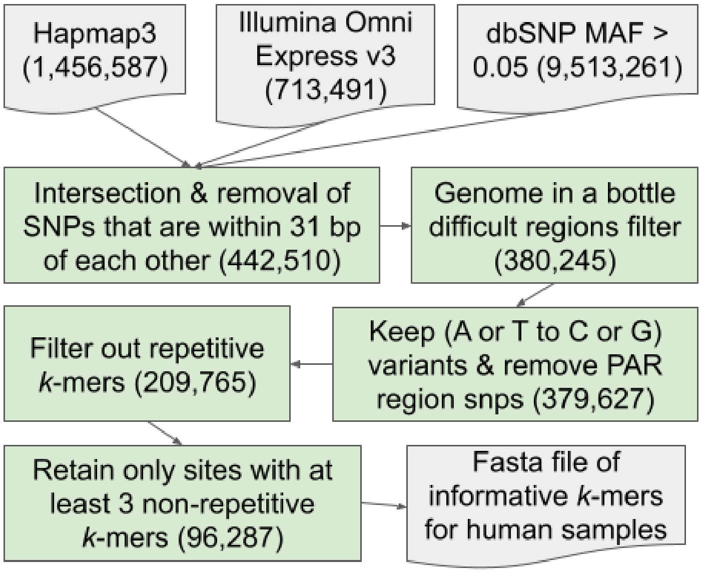

Figure 1. The selection method for informative human polymorphic sites used in *k*-mer counting based sample swap detection.

Generation PCA rotation matrices for human samples

In addition to variant sites sequences themselves, ntsm can optionally use population derived PCA rotational matrices which can help speed up comparisons of a large number of samples. We provide a python script that utilizes pandas [23] and scikit-learn [24] for those who wish to generate their own rotational matrices from a multiVCF file.

Using the multiVCF file from the 1000 Genome project [25], we generated a matrix of samples to our selected variant sites above (0 for homozygous A/T allele, 0.5 for heterozygous alleles, and 1 for homozygous C/G alleles). This is followed by normalizing the matrix by the standard deviation for each site and we keep the normalization vector in a file. This sample to variant matrix undergoes decomposition into principal components (Figure 2), though instead of being concerned with the principal component values themselves, we are primarily interested in keeping the rotational matrices for a number of the most significant components, and using them to project the new sequences onto.

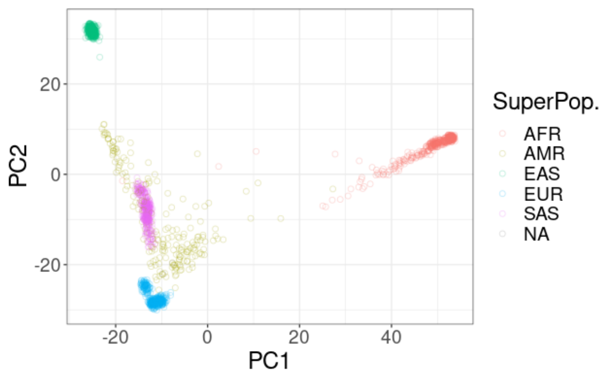

Figure 2. A population PCA of the first 2 components using the variant human sites selected and the 1000 Genomes Project multiVCF dataset. Each color represents a different labeled superpopulation group.

Implementation Details

Variant *k*-mer Counting

Paired variant sequences (1 file for C/G allele and 1 file for A/T variants) are stored in fasta files before being loaded. These alleles are then broken into *k*-mers and hashed using an invertible hash function into a hash table [26]. A sliding *k*-mer window for each allele is used, to provide redundancy to compensate for sequencing errors. Input sequences in fastq format are then read, broken in *k*-mers and also hashed [27], then subsequently checked for existence in the hash table. If they exist, then the occurrence count for that *k*-mer increments by one.

Optionally, the total number of *k*-mers that match a site can be used as criteria for early termination to save computational time, as only fairly low coverage is needed to perform accurate classification. For each allele, only the highest count of the sequence is outputted in the results counts file. The counts are a simple tab-separated (TSV) file with 3 columns for the site identifier, allele A/T count and allele C/G count. Multiple files and threads can be used on the same instance of the counting tool for speed, or run separately on different files and later merged.

Sequencing error rate estimation

Error rate can inform the user of the viability of the dataset and inform the user as to why downstream applications may be performing poorly. In our case, it may help explain why a sample swap signal is weak.

To estimate the error rate our tool records the total count of all *k*-mers seen t and the total number of *k*-mers matching to our set of *k*-mers m. To relate these values to each other, we need to know when no error is present the expected number of *k*-mers n. Assuming that the

data is randomly sampled from the genome, we can find the expected value of *n* given the diploid genome size *g* and the total number of distinct *k*-mers within our set of *k*-mer *d* with the following formula:

$$\hat{n} = \frac{td}{g}$$

Using *n*, we can use maximum likelihood estimation [28] (MLE) to derive the estimate the expected similarity *p*:

$$\mathcal{L}(p) = \left(p^k\right)^m \left(1 - p^k\right)^{n-m} = p^{km}\left(1 - p^k\right)^{n-m}$$

Working in log space will make our MLE derivation easier,

$$\log \mathcal{L}(p) = m \cdot \log\left(p^k\right) + (n - m) \cdot \log\left(1 - p^k\right)$$

Thus,

$$\frac{\partial \log \mathcal{L}(p)}{\partial p} = \frac{km}{p} - \frac{n - m}{1 - p^k} \cdot kp^{k-1} = \frac{k \cdot \left[m(1 - p^k) - (n - m)p^k\right]}{p\left(1 - p^k\right)}$$

The maximum likelihood estimate of *p* is obtained when $\partial \log \mathcal{L} / \partial p = 0$. Thus,

$$0 = \frac{k \cdot \left[m - mp^k - np^k + mp^k\right]}{p\left(1 - p^k\right)}$$

$$0 = m - np^k$$

Finally, similarity is formulated as:

$$\hat{p} = \left(\frac{m}{n}\right)^{1/k}$$

Error rate is merely the inverse of similarity,

$$ErrorRate = 1 - \left(\frac{m}{n}\right)^{1/k}$$

We note that this formulation largely holds true for mismatch and small indel error types, however when large indels are introduced this formulation can become less accurate depending on how one defines the ground truth alignment used to calculate the sequence error rate.

Similarity score for detecting sample swaps

Rather than computing genotypes for each site, our method directly uses the counts of each allele derived from k-mer counts. Our score is derived using likelihood ratio test as a basis with 2 models assuming that either the samples are independent or if they are the same sample. To minimize the effect of missing data due to low coverage, in each pairwise analysis we remove sites with missing counts for both alleles. The score is further modulated to be extra conservative, lowering the confidence of our result when coverage of the dataset is low.

To start, we need the likelihood of one sample. Suppose there are N sites. For a sample at site i, we observe x_{ia} count of allele a, where $a \in \{1, 2\}$ for the two alleles at each site i. Let p_{ia} be the probability of observing allele a at site i. Then the probability of the data **x** is:

$$\mathcal{L}(\mathbf{p}) = P(\mathbf{x}|\mathbf{p}) = \prod_{i=1}^{N} \prod_{a=1}^{2} p_{ia}^{x_{ia}}$$

The log-likelihood is

$$\log \mathcal{L}(p) = \sum_{i} \sum_{a} x_{ia} \log p_{ia}$$

The max-likelihood (ML) estimate of p_{ia} is

$$\hat{p}_{ia} = \frac{x_{ia}}{x_{i1} + x_{i2}}$$

Next, for a log-likelihood ratio test, we need to compare two models:

Model 1: two samples are independent
Let $L^{(1)}$ be the likelihood of sample 1 and so it is with sample 2. The total likelihood is $L^{(1)} \cdot L^{(2)}$.

Model 2: two samples are the same
In this case, we can merge all counts. Let

$$x_{ia}^{(*)} = x_{ia}^{(1)} + x_{ia}^{(2)}$$

Then the probability of the two sample is

$$P^{(*)} = \prod_{i=1}^{N} \prod_{a=1}^{2} \left[p_{ia}^{(*)}\right]^{x_{ia}^{(*)}}$$

The ML estimate of $p_{ia}^{(*)}$ is

$$\hat{p}_{ia}^{(*)} = \frac{x_{ia}^{(*)}}{x_{i1}^{(*)} + x_{i2}^{(*)}}$$

and the log-likelihood is

$$\log \mathcal{L}^{(*)}(p) = \sum_{i} \sum_{a} x_{ia}^{(*)} \log p_{ia}^{(*)}$$

Using the two models proposed previously we can input the results into the Log-likelihood ratio test [29]. The log likelihood ratios can be used to compute a robust score metric which can determine if two samples are of the same origin. The log-likelihood formulation is as follows:

$$\lambda_{\mathrm{LR}} = -2 \log \frac{\mathcal{L}^{(*)}}{\mathcal{L}^{(1)} \cdot \mathcal{L}^{(2)}}$$

We scale by the number of non-zero sites considered N and the coverage c_1 and c_2 of both samples. The c_1 and c_2 are included in the formulation to reduce our confidence in the results when the coverage is low (Supplementary Figure S1), though is empirically modulated with the skew parameter s (default 0.2). The final score formulation is as follows:

$$\lambda_{\mathrm{LR}}(c_1 c_2)^s / N$$

This score metric effectively accounts for lower confidence of the results when low coverage data is used.

PCA-based spatial-index for fast sample screening

Naively comparing samples is an all-to-all operation (*i.e.* $O(n^2)$), in which even state-of-the-art methods such as Somalier perform when finding similar samples. Indeed, one of the key novel innovations in Somalier was the use of genome sketches to minimize the time spent on each comparison, which is admittedly extremely fast. Here we opt for a more sensitive approach that utilizes count information, which we cannot easily collapse into a sketch. This results in a notably slower single element comparison time, however the overall time complexity is still quadratic if a naive approach is performed, so any performance gains made through increasing the efficiency of pairwise operations has a limit.

If many samples are being compared, we can speed up analysis by optionally combining the concept of population level PCA analysis [30] with a spatial index data structure called a kd-tree [31], with ntsm utilizing the nanoflann implementation of kd-trees [32]. Our method of generating a population PCA is mentioned in a previous section and we provide 20 rotational matrices for the human sites. At comparison time for each sample we take the variant sites and project them onto this PCA based on an existing population structure and then use a kd-tree to index them. Using a euclidean search radius in multidimensional space, we can then select the samples that occur in the local neighborhood of the sample being tested to minimize the number of comparisons being performed. We note that in order for this method to work the data must be of high quality, missing very few sites and have very accurate allele frequencies. Thus, our implementation uses the various criteria to determine if a sample is safe to use or must undergo a large radius search or even an exhaustive search.

Our search radius in this multidimensional space is determined by 2 properties of the data - the sequence error rate (estimated via method above), and the percent of missing sites (sites with a count less than the minimum count threshold). For the first radius (default = 2), only samples with a missing site percent less than 1% and an error rate less than 1% are permitted. For the second radius (default 15) a missing site percent greater than 30% is required. Finally if the data fails all of these conditions, an exhaustive search between all pairs is performed.

Calculating relatedness

Our method for computing relatedness largely borrows from the exact method described in the Somalier publication [11] which compensates for loss of heterozygosity seen in many tumor samples. Our implementation uses *k*-mer counts to create rough genotyping calls and omit

missing sites from the relatedness calculation. In our counts to make genotyping calls we filter *k*-mer occurrences less than two, to compensate for *k*-mers induced by sequencing errors.

Results

Validation of error rate estimation

Sample swap detection works better when sequence data is largely free from errors. However, though sequencing error rate can be broadly estimated by type of technology used, a sequence based estimate of the error rate can be invaluable to troubleshoot why some samples may have stronger or weaker or associations than expected. In addition, error rate is used when screening samples on whether our PCA based index can be used and autodetection of this helps simplify the user experience.

To measure the accuracy of our error rate estimation, our ground truth was based on the alignments to the CHM13 T2T reference genome [33]. We chose this effectively haploid genome to minimize any over estimation of error due using alignment to a reference as the ground truth. We used real Illumina, Pacbio Hifi and ONT data for CHM13 in addition to simulated data using wgsim [34] and PBSIM [35] at differing error rates. Error rate for real data is defined after alignment and we used the gap collapsed error rate (*i.e.* gap collapsed sequence identity [36]) metric in this case. Gap compressed error rate does not take into account error caused by gap lengthening but still takes into account indel and mismatches.

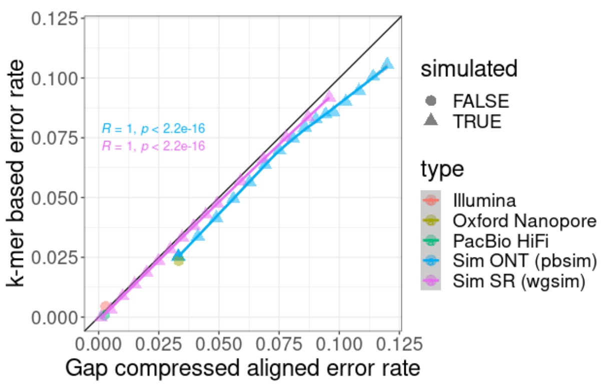

Figure 3. *k*-mer based error rate calculation vs Gap compressed align error rate ground truth on real and simulated CHM13 data onto the CHM13 T2T reference genome. The Pearson correlation coefficient (R) is provided showing high correlation between our k-mer based error

rate estimate to the gap compressed error rate computed from alignments.

We found that our estimates closely match the expected error rate (Fig. 3), though both the real and simulated ONT data was slightly underestimated on average, but not to a degree that makes the estimate unreliable. It is expected that error caused by indels, especially if long segments of these are present, would produce a lower calculated error rate as the formulation (see methods) for our error rate calculator assumes only mismatches can occur. That said, we expected the formulation's the short indels to contribute to the error calculation in a way very similar to mismatches which is reflected here. We note that for our method an estimated diploid genome size is needed, and for these tests the default value used was 6.2 Gb. This value of course will differ if a genome with a different size genome is used.

PCA-based heuristic investigation

To evaluate the efficacy of our PCA-based method to reduce the number of pairwise comparisons that we perform, we use data from the HPRC (Supplementary Table S1), and run them with ntsm while providing a rotation matrix with normalization values. The properties of the data varied wildly between coverage and error rate providing comprehensive expected performance of our heuristics given different data types.

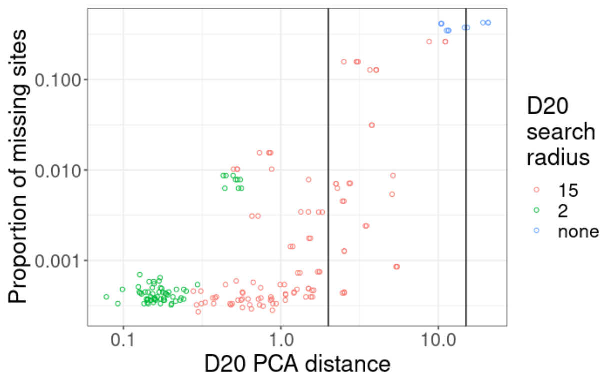

Figure 4. The PCA distance (top 20 PCs) between matching sample pairs vs the proportion of missing sites found in the dataset with the most missing sites. The colors (D20 search radius) correspond to the radius chosen by the algorithm for each sample pair based on the error rate and number of missing sites The vertical lines denote the radius thresholds of 2 and 15.

The number of missing sites greatly affects the performance of our heuristic, generally requiring a larger radius to search as the number of missing sites increase (Figure 4). We can measure the number of missing sites to apply thresholds prior to applying a search radius. The other variable determining the performance of our heuristic is the correctness of our genotype calls on

our data. This is influenced by both the coverage of the data and error rate of the data. We also use our error rate estimates to determine the radius to search. The coverage is largely a function of the missing sites and found it to be a better metric overall. Because of this, high coverage data generally requires a much smaller search radius (Supp. Figure S2).

When the *k*-mer coverage smaller dataset of a pair of matching samples >20x, we find that >57% of the samples are set to use a radius of 2, while at a coverage >30x, we find that >93% of the samples require a search radius of 2 (the remaining datasets at search radius of 15 being higher error nanopore data). At a search radius of 15 the number of pairs that will be considered will be <15% of the possible pairs, and at 2 the expected number of candidate pairs drops to <3% of possible elements on average (Figure 5).

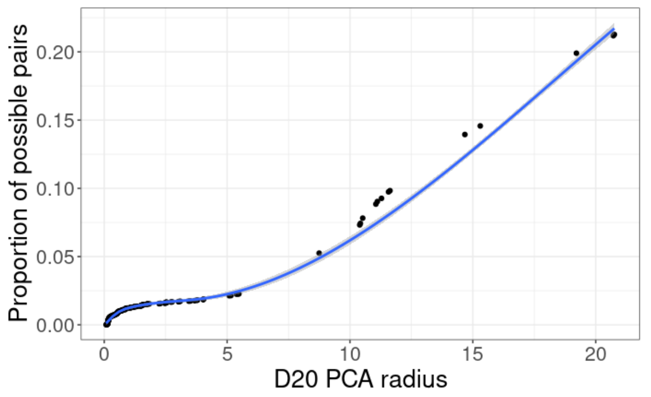

Figure 5. The proportion of all possible pairs evaluated if that radius within the PCA (first 20 PCs) was used in the search for all samples.

Comparisons to Somalier

To show the performance of ntsm, we sequence data from the HPRC [17] and a multiVCF file from the 1000 Genomes Project [25] featuring 3202 samples. We compare 39 samples with whole genome data from the HPRC which include sequencing data from Illumina, Pacbio HiFi, Hi-C, 10x Chromium, Strand-seq, and ONT platforms (Supplementary Table S1). As we expected ONT data to be the most difficult datatype to analyze, we chose only samples that had complementary data of this type in the analysis.

Sensitivity and Specificity of Sample Swaps

Unlike ntsm, Somalier does not provide an automated means of determining what should be considered similar enough to consider it the same sample. However, it does provide relatedness metrics that can easily be used to threshold samples showing high degrees of relatedness and thus similarity. To determine a threshold, we ran the full coverage of all the HPRC sample datasets (including all available data types) mentioned above for Somalier and manually picked a threshold that provided good separations between samples with the same origins from those with different origins (Figure 6). We determined that a relatedness value of 0.667 seemed to be

a good threshold for Somalier while still maintaining a high sensitivity. As mentioned in the methods section, ntsm uses a log-likelihood based score to separate samples with same origins from those with different origins, where the default value for this threshold is 0.5.

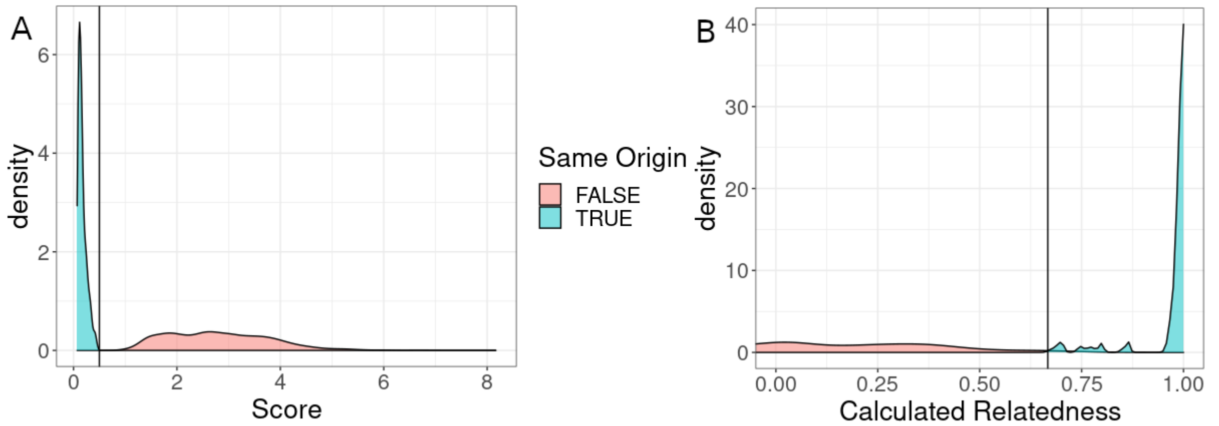

Figure 6. Distributions of metrics (score for ntsm (A) and relatedness for Somalier (B)) used for determining if two samples are similar. Horizontal lines are the thresholds used to determine the same sample status in later parts of the comparison (Relatedness > 0.67 for Somalier and Score < 0.5 for ntsm, respectively).

Next we randomly subsampled each dataset at different fold coverages (from 0.5x to 20x) and proceeded to see how each tool performed on this lower coverage data. We found that both tools are capable of detecting whether or samples have the same sample of origin at coverages higher than 5x, however Somalier struggled when attempting to match samples at coverage lower than 5x, producing a much higher number of false positive and negative pairs in the output (Figure 7). At sub-1x coverage, even ntsm struggled with detecting samples with the same origin, though not to the degree that Somalier struggled. In general, ntsm benefits from higher coverage as well, generating higher scores (i.e. more confident results) for unmatched samples (Supplementary Figure S1.).

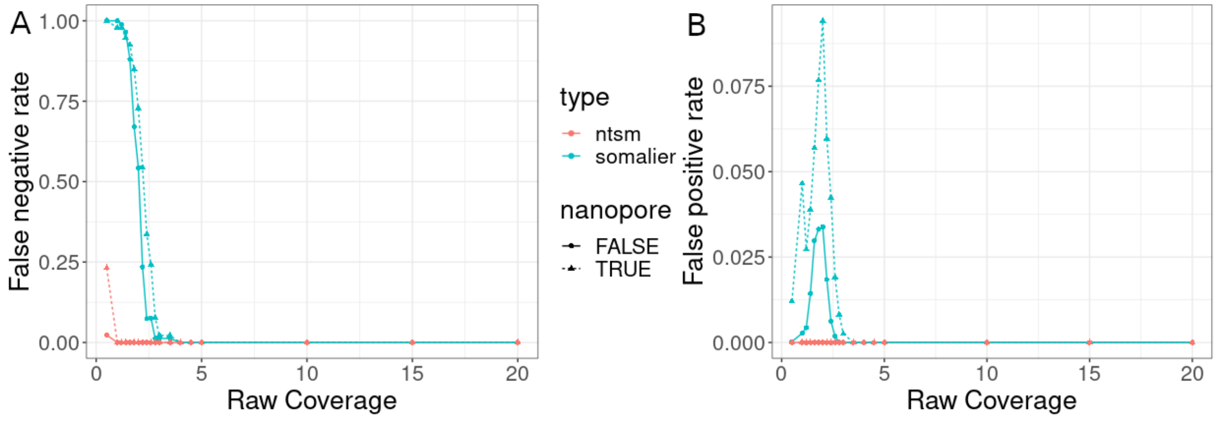

Figure 7. Effects of dataset coverage on classification performance on Somalier and ntsm. A. The false negative rate (FNR) of Somalier and ntsm at varying raw dataset coverage. B. The false positive rate (FPR) of Somalier and ntsm at varying raw dataset coverage. Nanopore combined with other datasets is shown separately due to the high error rate of the former. The result for ntsm are the same regardless of if the PCA heuristic is used or not (no effect on sensitivity and specificity in this case).

Memory and time comparisons

Workflow for processing raw read data for Somalier and ntsm can be thought of in 2 major steps: (1) Per sample read processing to obtain variant site information and (2) Joint sample variant site information all-to-all comparisons to obtain similarity. In Somalier, step 1 entails taking raw reads, aligning them to a reference (using aligners like bwa or minimap2, depending on the read type), sorting and indexing them (using samtools), and finally extracting the variant information for specific sites into a bitvector (somalier extract). The computational resources required for this step are dominated by the alignment stage. Finally, step 2 takes each extracted bitvector and runs pairwise comparison between all of them to obtain relatedness. While in ntsm, step 1 entails taking raw reads and counting *k*-mers for specific sites (ntsmCount). These count files are then compared (ntsmEval) using the count information directly in step 2.

k-mer counting (ntsm) vs alignment (somalier)

One of the primary benefits of ntsm is bypassing the alignment requirement that other tools require. However, the alternative we must perform is *k*-mer counting, which, though is fairly resource frugal, is not free. To determine the relative resource cost counting takes in comparison to alignment, we took equal coverage subsamples (2x) of our datasets (Supplementary Table S1) and ran the subsets with ntsm and each alignment tool we used to generate alignments needed for Somalier.

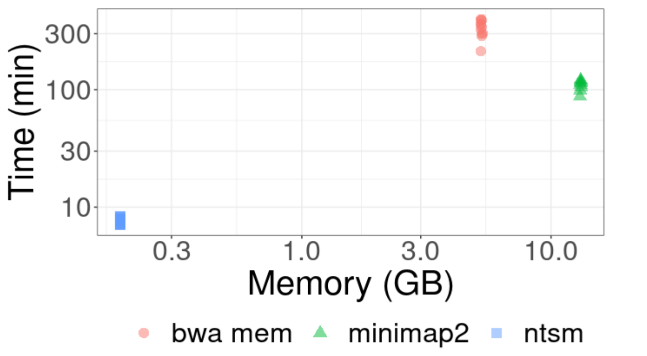

Figure 8. Peak memory and wallclock time comparisons of *k*-mer counting vs alignment both using single thread.

We then measured the time and memory needed for each tool used (Figure 8). We found that ntsm ran at an average of ~8 minutes, orders of magnitude less than bwa mem and minimap2 at ~1.9 CPU hours and 5.9 CPU hours respectively. Memory usage is low because we are only counting a very small specific subset of *k*-mers. Note that we did not include sorting or indexing time in this analysis as we hoped to illustrate that even without this in our comparison *k*-mer counting was still much less resource intensive. Also sorting can partially be run in parallel with alignments as reads are streamed.

Sample comparison process

As mentioned in the method section, comparison of all samples with each other is naively an all-to-all operation and thus a quadratically scaling operation. For most studies this may still be a trivial concern but as larger and larger studies are considered, this can become an increasingly expensive consideration. To observe the computational expense of this process we created simulated counts files (for ntsm) and bit vector files (for Somalier) for the samples found in the 1000 Genomes VCF and measured time and memory used to detect sample swaps between subsamples at different sizes. Though most of the time reads will be used as input, both Somalier and ntsm can use multiVCF files to generate variant site information. This enables the users to compare reads files to existing vcf files but would likely not be the primary usage of these tools though benefits us here enabling us to see demanding a large number of comparisons can be.

As expected we observed that pairwise comparison time scaled quadratically (Figure 9, Supp. Table S2), even in Somalier, albeit with each individual comparison being orders of magnitude faster than our method. Somalier, utilizing a bitvector based comparison method is much more optimized for speed than our count based method. However for the sample comparison stage ntsm is capable of using more than one thread which can help close the gap in speed. When using 16 threads with ntsm in PCA mode we were able to remain competitive at 32 seconds compared to 15 seconds respectively on 3202 samples. For sample comparison, Somalier is currently unable to use multiple threads. Additionally, we observe here that our PCA-based screening approach actually may scale less than quadratically relative to the number of samples though it requires high coverage and low error rate data to perform this way reliably. Memory usage is largely linear relative to the data (Supp. Figure S3) as expected and there is no additional memory overhead using our PCA-based method.

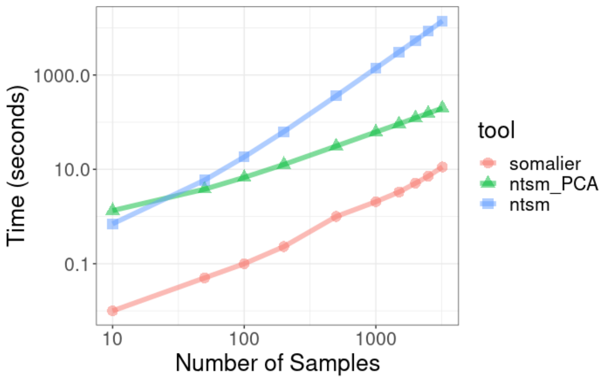

Figure 9. Wall clock time comparisons of somalier vs ntsm with and without PCA-based screening. A multiVCF file for 3202 samples from the 1000 Genome project using 1 thread.The results shown here only include timing after conversion to input type needed for each tool (using `ntsmVCF` for ntsm and ‘somalier extract` for somalier).

k-mer based relatedness calculation

Relatedness computation is included for all sample pairs reported. To test our relatedness estimation method we took Pacbio HiFi datasets of the parent (HG003, HG004) child (HG002) trio. Both ntsm and Somalier correctly computed the relatedness we expected; that is, parents remained unrelated (0%), while child samples showed 50% relatedness to its parents and with 100% relatedness to a technical replicate to itself (Figure 10)

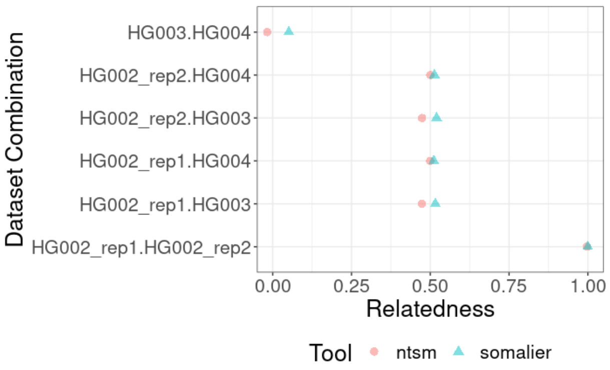

Figure 10. Comparison of Somalier and ntsm computed relatedness of parent child trio (HG002, HG003, HG004) from Pacbio HiFi sequencing reads. Two independent sequencing runs of HG002 are used here.

Though the similarity values largely agree between ntsm and Somalier, there are minor differences between our calculations. These differences likely primarily stem from the fact that

Somailer and ntsm use different variant sites and that we use *k*-mer counts to create genotyping calls and omit missing sites from the relatedness calculation. The differences in our tool are likely due to the number sites used by default by somalier (total of 17,766 sites), and the sites we selected (see methods, total of 96,287 sites). In addition, some of the differences in our relatedness calculation are a consequence of omitting missing sites, which Somalier is unable to perform as it computes similarity using bit vectors of a uniform length.

To gain a more comprehensive view on the accuracy of our relatedness calculations we checked the quality of our relatedness estimates using samples shown to be related in 1000 Genomes cohorts in addition supplemented with samples with at least 20x coverage sequence coverage of various sequencing data types (Supp. Table 1). We found that overall ntsm produces a relatedness metric closer and more tightly grouped to the expected value based on the pedigree (Figure 11). We note however this trend does not hold true for relatedness estimates involving a nanopore dataset, showing that ntsm calculates relatedness conservatively when it comes to data originating from the same sample.

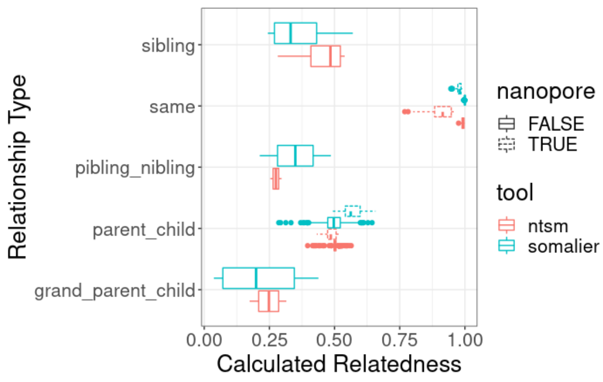

Figure 11. Comparison of known related samples in 1000 Genomes cohort, supplemented with samples with ~20x coverage sequence coverage of various sequencing data types (Supp. Table 1). Relatedness calculations involving a Nanopore dataset are shown separately.

Discussion

Here we have described NTSM, a tool designed for sample swap detection in QC contexts. The major benefits are that it uses resource frugal counts of specific *k*-mers rather than alignments decreasing overall computational costs and its capability to robustly function independent of sequencing technology type with high sensitivity on low coverage data. For large scale multi sample comparisons, we utilize a novel PCA-based spatial index heuristic screening method that greatly reduces the computational cost of comparing samples by reducing the number of candidates to compare. Overall over previous alignment based methods, we believe that ntsm

could be an effective upstream tool in large scale studies, enabling robust QC and reducing the chances of error as studies become larger and incorporate more diverse sequencing data types.

Reference-based vs reference-free

Our tool, unlike generic k-mer comparison methods like MASH [3], requires a set of variant sites to then derive the non-repetitive sets of *k*-mers. We recognize that a tool that uses reference-free information like raw *k*-mer spectrum information would be more desirable than our reference-based method using *k*-mers from a set of reference-derived sites. However, any generic reference-free method that uses a *k*-mer spectrum analysis approach or similar would require much higher coverage, larger sequence differences within between samples, and very low error rates and noise. Here, the reference/population based information provides the information needed for the high statistical power and robustness of intra-species sample swap identification. Finally, beyond statistical considerations, it is computationally trivial to consider only a subset of sequences existing or not than it is to index and compare the spectrum of sequences between two samples.

Validation and Comparison with Somalier:

We compared ntsm to Somalier [4], another state-of-the-art tool designed in part for sample swap detection, using data from the HPRC [17] as well as 1000 Genome project [25]. In particular we showed that on low coverage, and high error rate data, ntsm outperforms it in terms of sensitivity and specificity, able to correctly match all 137 samples to their corresponding samples with the same origin (184 matching pairs) with no false positives at even as low as 1x raw coverage. In addition, though not our goal, we found that ntsm also outperformed Somalier in estimating relatedness, producing relatedness estimates closer to the known pedigrees. Somalier utilizes alignments, and we showed that alignment operations were an order of magnitude slower than our count based algorithm.

Conceptually, our alignment-free method does not perform more accurate mapping than alignment, rather the power of our method is resultant from using count information to perform a joint statistical test that can compensate for missing data, while the alignment-free methodology enables faster analysis while maintaining adequate performance compared to alignment. Specifically, our unique application of sample swap detection lends itself to being able to select variant sites which do not contain repetitive *k*-mers (ensure high specificity) as only a subset of all informative variants are needed for this type of analysis. Finally, to maintain a high sensitivity, relatively small (19-mers) are used and can still detect useful *k*-mers in high error rate data.

When it comes to the speed of pairwise comparisons post alignment or counting, Somalier outperformed ntsm as Somalier uses fast bit vector based comparison, while we use a slower but more accurate count based statistical test. PCA-based spatial index heuristic helped reduce the time complexity of our method and saved orders of magnitude by reducing the number of comparisons, but is by is limited by the quality of the data. Somalier's pairwise comparisons outperformed even our PCA-based heuristic computationally in our tests, however we believe in

theory as somalier remains quadratic in complexity it may be possible to outperform it with our PCA-based heuristic.

We note that our we do not wish to overstate the computational cost of pairwise comparisons, as even with the full 3202 samples of the 1000 Genome Project the pairwise evaluation times for ntsm on a single thread were ~4 hours for ntsm, and ~3 mins for ntsm with our PCA heuristic and 11 seconds for Somalier. For perspective, if we had used raw illumina short reads rather than a VCF file, 3202 samples would take at over 5 CPU years to align with bwa mem as compared to k-mer counting in only 43 CPU days, assuming the average alignment time for each tool (Figure 8), a single thread, and 5x coverage (sufficient coverage to make sample swap determination for short reads in Figure 7). Of course parallelism can be applied in these cases but we intend to show that at present for most applications we expect the computational costs to be dominated by mapping rather than pairwise comparisons.

Conceptually, it is non-trivial to compare the contributions to computational costs of the read mapping stages versus the pairwise comparison stage, as the read mapping stages is mostly linear in time complexity while the pairwise comparison complexity is mostly quadratic in time complexity. Here we have reduced the computational cost of the mapping stage and increased sensitivity and specificity of finding sample swaps at the cost of a slower pairwise comparison speed. We believe if the goal is sample swap detection then ntsm would likely be the best choice due to its higher sensitivity and lower upfront mapping computational costs, but if the goal is computing comprehensive relatedness between all samples in a very large cohort of a multitude of already variant called data, then Somalier would be the superior practical choice due the speed of its pairwise comparisons.

Current Limitations

Though robust enough to compare most samples originating from different sequencing technologies, not all possible sequencing data types have been tested and may not work with our method. Our statistical tests were formulated with the assumption that the input roughly originates from a whole genome shotgun sample. We have not yet tested data types such as whole exome data [37], RNA-seq [38] or ChIP-seq [39]. The data types largely differ by the extreme coverage differences between sites and the fact that more specialized sets of sites (*i.e.* transcribed regions) would likely need to be selected.

Though our method can be adapted to work on more than just human genomes, our method currently assumes sites with two alleles with similar frequency. Thus, detecting sample swaps of non-diploid genomes using our method will require adaptations to the models we use, but we are optimistic that principle behind it (*i.e.* the use of population level allele frequency information and sequence coverage information) could be used to detect sample swaps in those instances.

Conclusions

As studies become larger and more complex, sample swaps in data are inevitable. This tool could become an integral part of upstream pipelines, robust enough to be agnostic of any sequencing technology or library preparation method. In addition, this will also reduce error when it comes to collaboration between labs and they will be able to easily match data originating from the same sample even if orthogonal sequencing technologies are used. Our novel PCA based spatial index heuristic opens the possibility of sub-quadratic comparison time complexity when comparing samples and shows its potential here, though in principle we believe the methodology can still be improved on especially when it comes to compensating for missing data. We believe our counting based alignment free methodology presented here has very little computational overhead and can readily be applied upstream to preexisting many data production pipelines.

Availability of supporting source code and requirements

Project name: ntsm
Project home page: https://github.com/JustinChu/ntsm
Operating system(s): Linux
Programming language: C++
Other requirements: TBD
License: MIT
BiotoolsID: ntsm
RRID: SCR_024994

Data Availability

All supporting data and materials are available in the GigaScience GigaDB database [40]. The GigaDB record has a bioinformatics notebook, called bioinformatics_notebook.txt (natch!).

List of abbreviations

bp: base pair; dbSNP: The Single Nucleotide Polymorphism Database; Pacbio HiFi reads: Pacific Bioscience High-Fidelity; HPRC: Human Pangenome Reference Consortium; indel: insertion deletion; MAF: Minor allele frequency; MLE: Maximum Likelihood Estimation; ONT: Oxford Nanopore Technology; PCA: Principal Component Analysis; QC: Quality Control; SNP: Single Nucleotide Polymorphism; T2T: Telomere to Telomere; tsv: Tab-separated values; VCF: Variant Call Format; VGP: Vertebrate Genome Project

Consent for publication

All the datasets used in this study are publicly available. All authors consent to publication of this manuscript.

Competing interests

The authors declare that they have no competing interests.

Funding

This work was supported by National Human Genome Research Institute Grants U01HG010961 and R01HG010040.

Authors' contributions

J.C. and H.L. conceived and designed the project; J.R. prototyped the methodology for the selection of variant sites; X.F. provided the initial methodology for performing PCA on variant sites; J.C. implemented ntsm, performed the evaluations and drafted the manuscript; all authors edited, read and approved the final manuscript.

References

1. Chu J, Sadeghi S, Raymond A, Jackman SD, Nip KM, Mar R, et al.. BioBloom tools: fast, accurate and memory-efficient host species sequence screening using bloom filters. *Bioinformatics*. 2014; doi: http://dx.doi.org/10.1093/bioinformatics/btu558.

2. Cornet L, Baurain D. Contamination detection in genomic data: more is not enough. *Genome Biol*. BioMed Central; 2022; doi: http://dx.doi.org/10.1186/s13059-022-02619-9.

3. Ondov BD, Treangen TJ, Melsted P, Mallonee AB, Bergman NH, Koren S, et al.. Mash: fast genome and metagenome distance estimation using MinHash. *Genome Biol*. 2016; doi: http://dx.doi.org/10.1186/s13059-016-0997-x.

4. Manichaikul A, Mychaleckyj JC, Rich SS, Daly K, Sale M, Chen WM. Robust relationship inference in genome-wide association studies. *Bioinformatics*. Bioinformatics; 2010; doi: http://dx.doi.org/10.1093/bioinformatics/btq559.

5. Bergmann EA, Chen B-J, Arora K, Vacic V, Zody MC. Conpair: concordance and contamination estimator for matched tumor–normal pairs. *Bioinformatics*. Oxford Academic; 2016; doi: http://dx.doi.org/10.1093/bioinformatics/btw389.

6. Wang PPS, Parker WT, Branford S, Schreiber AW. BAM-matcher: a tool for rapid NGS sample matching. *Bioinformatics*. Oxford Academic; 2016; doi:

http://dx.doi.org/10.1093/bioinformatics/btw239.

7. Schröder J, Corbin V, Papenfuss AT. HYSYS: have you swapped your samples? *Bioinformatics*. Oxford Academic; 2016; doi: http://dx.doi.org/10.1093/bioinformatics/btw685.

8. Lee S, Lee S, Ouellette S, Park W-Y, Lee EA, Park PJ. NGSCheckMate: software for validating sample identity in next-generation sequencing studies within and across data types. *Nucleic Acids Res*. Oxford Academic; 2017; doi: http://dx.doi.org/10.1093/nar/gkx193.

9. Pedersen BS, Quinlan AR. Who's Who? Detecting and Resolving Sample Anomalies in Human DNA Sequencing Studies with Peddy. *Am J Hum Genet*. Elsevier; 2017; doi: http://dx.doi.org/10.1016/j.ajhg.2017.01.017.

10. Javed N, Farjoun Y, Fennell TJ, Epstein CB, Bernstein BE, Shoresh N. Detecting sample swaps in diverse NGS data types using linkage disequilibrium. *Nat Commun*. Nature Publishing Group; 2020; doi: http://dx.doi.org/10.1038/s41467-020-17453-5.

11. Pedersen BS, Bhetariya PJ, Brown J, Kravitz SN, Marth G, Jensen RL, et al.. Somalier: rapid relatedness estimation for cancer and germline studies using efficient genome sketches. *Genome Med*. 2020; doi: http://dx.doi.org/10.1186/s13073-020-00761-2.

12. Bennett S. Solexa Ltd. *Pharmacogenomics*. Pharmacogenomics; 2004; doi: http://dx.doi.org/10.1517/14622416.5.4.433.

13. Branton D, Deamer DW, Marziali A, Bayley H, Benner SA, Butler T, et al.. The potential and challenges of nanopore sequencing. *Nat Biotechnol*. NIH Public Access; 2008; doi: http://dx.doi.org/10.1038/nbt.1495.

14. Belton J-M, McCord RP, Gibcus J, Naumova N, Zhan Y, Dekker J. Hi-C: A comprehensive technique to capture the conformation of genomes. *Methods*. NIH Public Access; 2012; doi: http://dx.doi.org/10.1016/j.ymeth.2012.05.001.

15. Zhang M, Zhang Y, Scheuring CF, Wu C-C, Dong JJ, Zhang H-B. Preparation of megabase-sized DNA from a variety of organisms using the nuclei method for advanced genomics research. *Nat Protoc*. Nature Publishing Group; 2012; doi: http://dx.doi.org/10.1038/nprot.2011.455.

16. Rhie A, McCarthy SA, Fedrigo O, Damas J, Formenti G, Koren S, et al.. Towards complete and error-free genome assemblies of all vertebrate species. *Nature*. 2021; doi: http://dx.doi.org/10.1038/s41586-021-03451-0.

17. Liao W-W, Asri M, Ebler J, Doerr D, Haukness M, Hickey G, et al.. A draft human pangenome reference. *Nature*. 2023; doi: http://dx.doi.org/10.1038/s41586-023-05896-x.

18. The International HapMap Consortium. The International HapMap Project. *Nature*. Nature Publishing Group; 2003; doi: http://dx.doi.org/10.1038/nature02168.

19. Illumina: The Omni Family of Microarrays. https://www.illumina.com/Documents/products/datasheets/datasheet_gwas_roadmap.pdf Accessed 2023 Oct 27.

20. Smigielski EM, Sirotkin K, Ward M, Sherry ST. dbSNP: a database of single nucleotide polymorphisms. *Nucleic Acids Research*. 2000; doi: http://dx.doi.org/10.1093/nar/28.1.352.

21. Zook JM, Catoe D, McDaniel J, Vang L, Spies N, Sidow A, et al.. Extensive sequencing of seven human genomes to characterize benchmark reference materials. *Sci Data*. 2016; doi: http://dx.doi.org/10.1038/sdata.2016.25.

22. Li H, Durbin R. Fast and accurate short read alignment with Burrows–Wheeler transform. *Bioinformatics*. Oxford Academic; 2009; doi: http://dx.doi.org/10.1093/bioinformatics/btp324.

23. The pandas development team. pandas-dev/pandas: Pandas. Zenodo; 2023; doi: http://dx.doi.org/10.5281/ZENODO.3509134.

24. Garreta R, Moncecchi G. Learning Scikit-Learn: Machine Learning in Python. Packt Pub Limited;

25. 1000 Genomes Project Consortium, Auton A, Brooks LD, Durbin RM, Garrison EP, Kang HM, et al.. A global reference for human genetic variation. *Nature*. 2015; doi: http://dx.doi.org/10.1038/nature15393.

26. Goetghebuer-Planchon T: Website. https://github.com/Tessil/robin-map

27. Wang T: Integer Hash Function. http://web.archive.org/web/20071223173210/http://www.concentric.net/~Ttwang/tech/inthash.htm Accessed 2023 Sep 8.

28. Fisher RA. On the mathematical foundations of theoretical statistics. *Philos Trans R Soc Lond*. The Royal Society; 1922; doi: http://dx.doi.org/10.1098/rsta.1922.0009.

29. Wilks SS. The large-sample distribution of the likelihood ratio for testing composite hypotheses. *Ann Math Stat*. Institute of Mathematical Statistics; 1938; doi: http://dx.doi.org/10.1214/aoms/1177732360.

30. Patterson N, Price AL, Reich D. Population structure and eigenanalysis. *PLoS Genet*. 2006; doi: http://dx.doi.org/10.1371/journal.pgen.0020190.

31. Bentley JL. Divide and Conquer Algorithms for Closest Point Problems in Multidimensional Space.

32. Blanco-Claraco JL: GitHub - jlblancoc/nanoflann: nanoflann: a C++11 header-only library for Nearest Neighbor (NN) search with KD-trees. GitHub. https://github.com/jlblancoc/nanoflann Accessed 2023 Oct 30.

33. Nurk S, Koren S, Rhie A, Rautiainen M, Bzikadze AV, Mikheenko A, et al.. The complete sequence of a human genome. *Science*. 2022; doi: http://dx.doi.org/10.1126/science.abj6987.

34. Danecek P, Bonfield JK, Liddle J, Marshall J, Ohan V, Pollard MO, et al.. Twelve years of SAMtools and BCFtools. *Gigascience*. Oxford Academic; 2021; doi: http://dx.doi.org/10.1093/gigascience/giab008.

35. Ono Y, Asai K, Hamada M. PBSIM2: a simulator for long-read sequencers with a novel generative model of quality scores. *Bioinformatics*. Oxford Academic; 2020; doi: http://dx.doi.org/10.1093/bioinformatics/btaa835.

36. Li H: On the definition of sequence identity. https://lh3.github.io/2018/11/25/on-the-definition-of-sequence-identity Accessed 2023 Oct 27.

37. Albert TJ, Molla MN, Muzny DM, Nazareth L, Wheeler D, Song X, et al.. Direct selection of human genomic loci by microarray hybridization. *Nat Methods*. Nat Methods; 2007; doi: http://dx.doi.org/10.1038/nmeth1111.

38. Wang Z, Gerstein M, Snyder M. RNA-Seq: a revolutionary tool for transcriptomics. *Nat Rev Genet*. 2009; doi: http://dx.doi.org/10.1038/nrg2484.

39. Johnson DS, Mortazavi A, Myers RM, Wold B. Genome-wide mapping of in vivo protein-DNA interactions. *Science*. Science; 2007; doi: http://dx.doi.org/10.1126/science.1141319.

40. Chu J, Rong J, Feng X, Li H. Supporting data for "ntsm: an alignment-free, ultra low coverage, sequencing technology agnostic, intraspecies sample comparison tool for sample swap detection." GigaScience Database 2024; doi: http://dx.doi.org/10.5524/102521.

Supplement

Read sequence dataset information

Sample ID	Hi-c	Nanopore	Strand seq	Illumina	PacBio HiFi	10X
HG002	84.13	82.88	6.77	30.51	39.64	-
HG00438	82.2	39.08	-	30.33	29.19	-
HG005	-	103.13	-	47.02	44.01	-
HG00621	83.7	30.06	-	29.36	-	-
HG00673	86.09	32.83	-	28.7	-	-
HG00733	57.12	62.31	-	30.89	32.19	-
HG00735	72.78	29.35	-	30.04	-	-
HG00741	68.35	41.96	-	31.1	-	-
HG01071	68.6	37.71	-	31	-	-
HG01106	64.23	38.8	-	31.17	-	-
HG01109	19.01	58.78	-	30.56	30.18	59.76
HG01123	52.97	24.47	1.65	-	37.15	-
HG01175	68.64	42.14	-	29.2	34.83	-
HG01243	26.97	50.57	-	29.71	33.16	53.05
HG01258	62.85	19.76	-	31.49	-	-
HG01358	53.63	14.67	2.5	31.5	-	-
HG01361	46.74	30.67	-	32.64	-	-
HG01891	41.53	34.35	1.57	30.6	-	-
HG01928	68.75	22.85	-	32.66	-	-
HG01952	61.03	29.18	-	31.26	-	-
HG01978	85.89	36.74	-	33.56	-	-
HG02055	28.8	52.38	-	30.65	37.39	58.32
HG02080	28.78	47.63	-	31.92	32.89	45.07
HG02148	63.77	17.39	-	29.09	-	-
HG02257	50.71	18.43	3.98	31.92	-	-
HG02486	38.47	27.02	1.96	-	-	-
HG02559	48.27	22.59	-	-	-	-
HG02572	115.82	14.59	-	30.37	-	-
HG02622	52.18	13.56	-	33.3	-	-
HG02630	54.73	18.36	-	33.31	-	-
HG02717	72.3	25.38	-	32.68	-	-
HG02886	56.86	30.7	-	34.2	-	-
HG03098	23.01	44.57	-	30.66	33.55	57.83
HG03453	60.4	10.29	-	29.93	-	-
HG03471	58.84	27.46	-	-	-	-
HG03492	30.48	40.36	-	29.19	31.61	56.05
HG03516	49.44	40.25	-	28.65	-	-
HG03540	52.88	27.05	-	33.81	-	-
HG03579	69.9	14.8	-	29.06	-	-

Supplementary Table S1. HPRC sequencing datasets used in analysis. Each column after the first represents the aligned coverage (either using minimap2 or bwa mem) of each data type available for analysis.

size	ntsm Time (seconds)	ntsm Memory (kbytes)	ntsm_PCA Time (seconds)	ntsm_PCA Memory (kbytes)	Somalier Time (seconds)	Somalier Memory (kbytes)
10	0.7	37092	1.34	71140	0.01	4736
50	5.91	97260	3.8	131308	0.05	14848
100	18.31	172532	6.74	206324	0.1	31104
200	63.01	322812	12.68	356732	0.23	70144
500	361.27	774172	31.17	808356	1	239360
1000	1395.81	1526716	61.85	1559388	2.06	395136
1500	3079.83	2279156	91.9	2311780	3.31	541696
2000	5400	3031456	123.82	3064028	5.09	702464
2500	8476	3783956	154.57	3816724	7.16	894592
3202	13988	4840296	199.3	4872812	11.24	1149824

Supplementary Table S2. Time and memory of pairwise sample comparison of ntsm, ntsm with our PCA heuristic, and Somalier on the 1000 Genomes dataset.

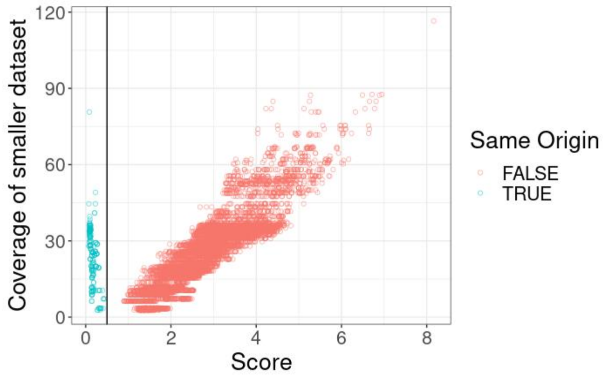

Supplementary Figure S1. Comparison of score and *k*-mer coverage on samples from the same origin and not. Confidence in distinguishing samples increases as coverage increases, reflected in high scores the more distant they are.

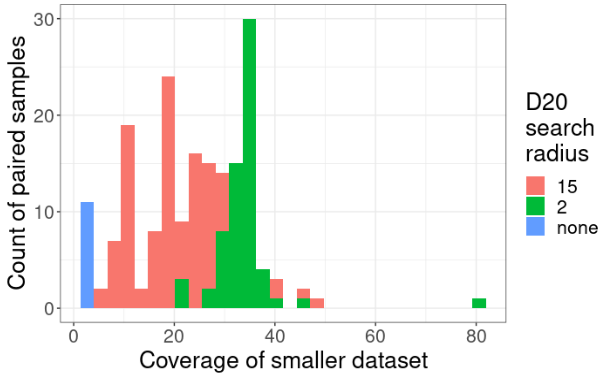

Supplementary Figure S2. The *k*-mer coverage of the smaller sample in a matching sample pair and the number of sample pairs that use a specific search radius.

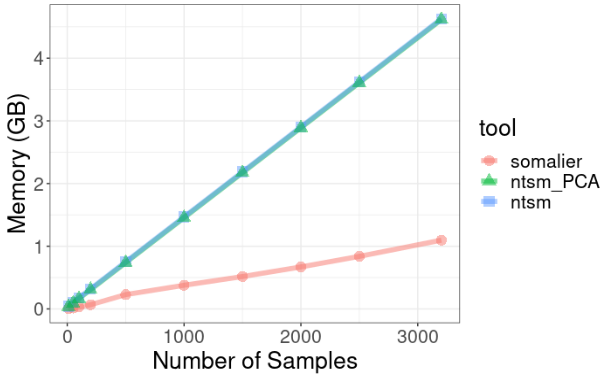

Supplementary Figure S3. The memory of somalier vs ntsm using with and without PCA-based prescreening, processing similarity between 3202 samples from the 1000 Genome project.

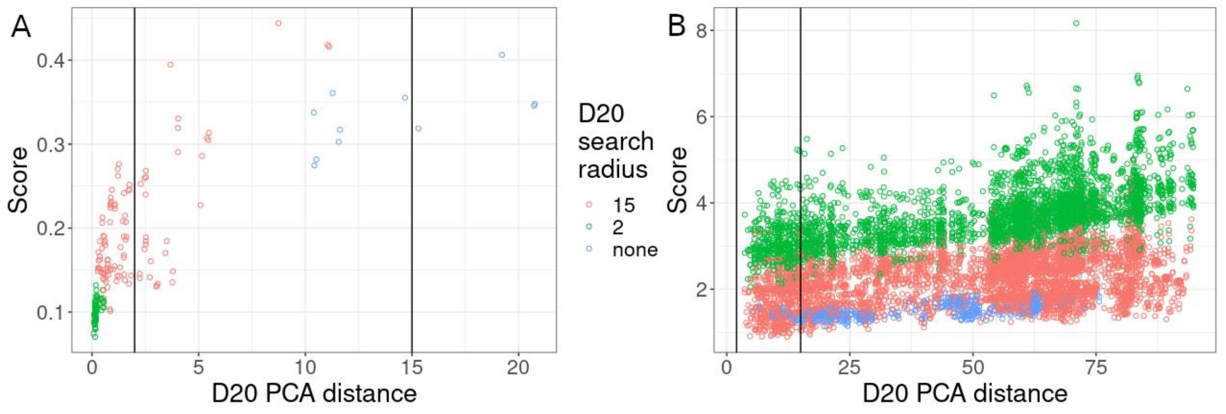

Supplementary Figure S4. PCA distance (20 dimensions) vs Score for same samples that match (A) and samples that do not match (B) from the HPRC read sets (Supplementary table S1). Vertical lines denote PCA search radii used when finding candidates for score computation.

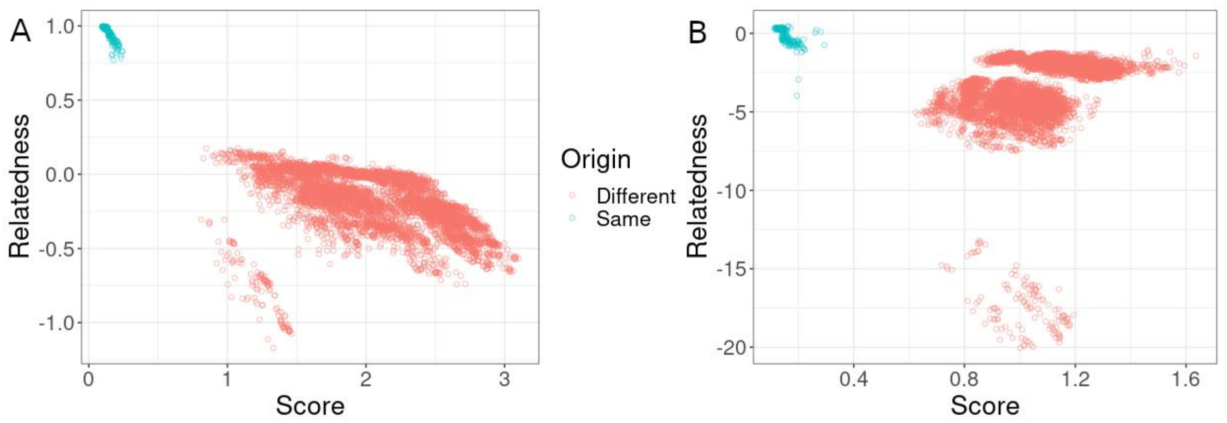

Supplementary Figure S5. Score vs Computed Relatedness for same samples that match and samples that do not match from the HPRC read sets (Supplementary table S1) subset to 20x (A) and 5x (B) raw coverage.

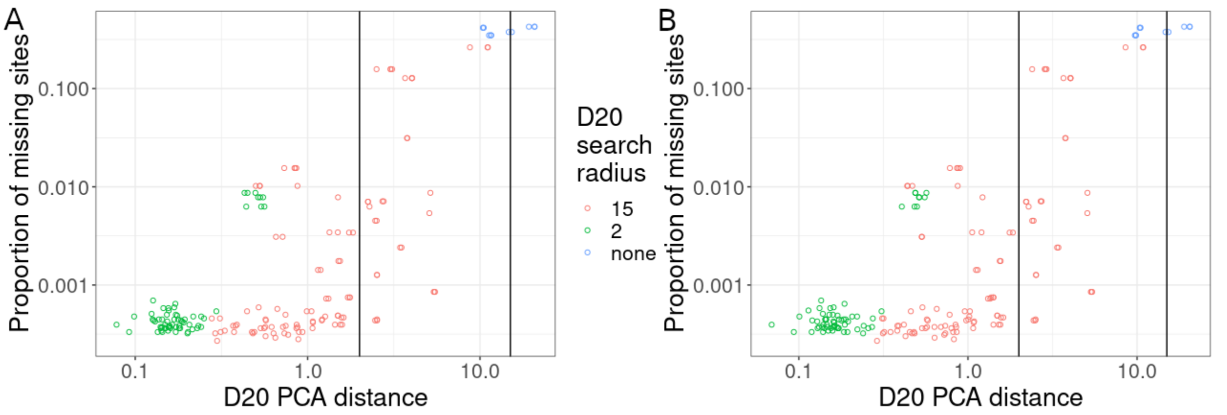

Supplementary Figure S6. PCA distance (first 20 PCs) vs missing sites and search radius imposed by the ntsm algorithm on HPRC datasets with a PCA created from the 1k genomes VCF (A), and a PCA created from 1k genome VCF minus any sample IDs shared in the HPRC dataset (B). The distances calculated differ slightly but are largely the same, owing to the similar general population structures even if some samples are removed. These plots correspond to Figure 4 in the main text.

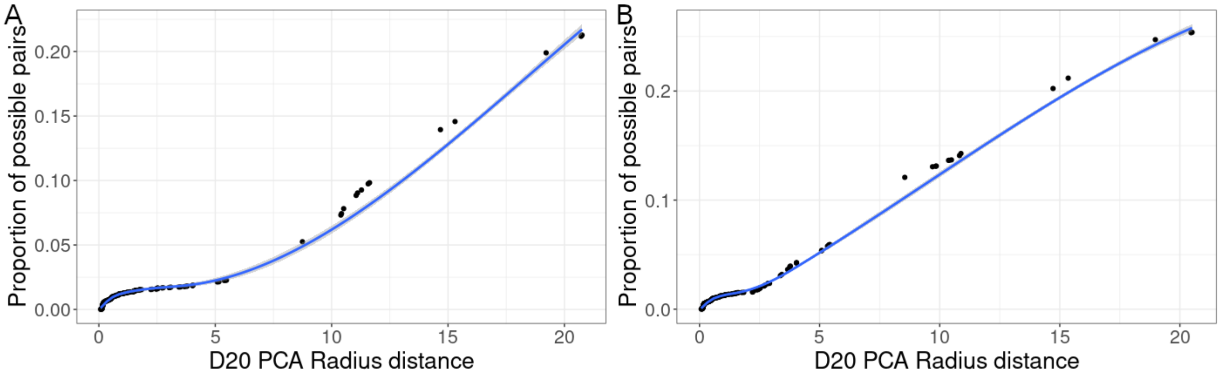

Supplementary Figure S7. The proportion of all possible pairs evaluated if that radius within the PCA (first 20 PCs) was used in the search for all samples with a PCA derived from all samples within the 1k genomes VCF (A) and a PCA created from 1k genome VCF minus any sample IDs shared in the HPRC dataset (B). The shape of the plot at lower radii seems to perform slightly better without the HPRC samples IDs removed but the overall trend in terms of search space reduction still remains the same. These plots correspond to Figure 5 of the main text.

Click here to access/download;Figure;Figure1.png

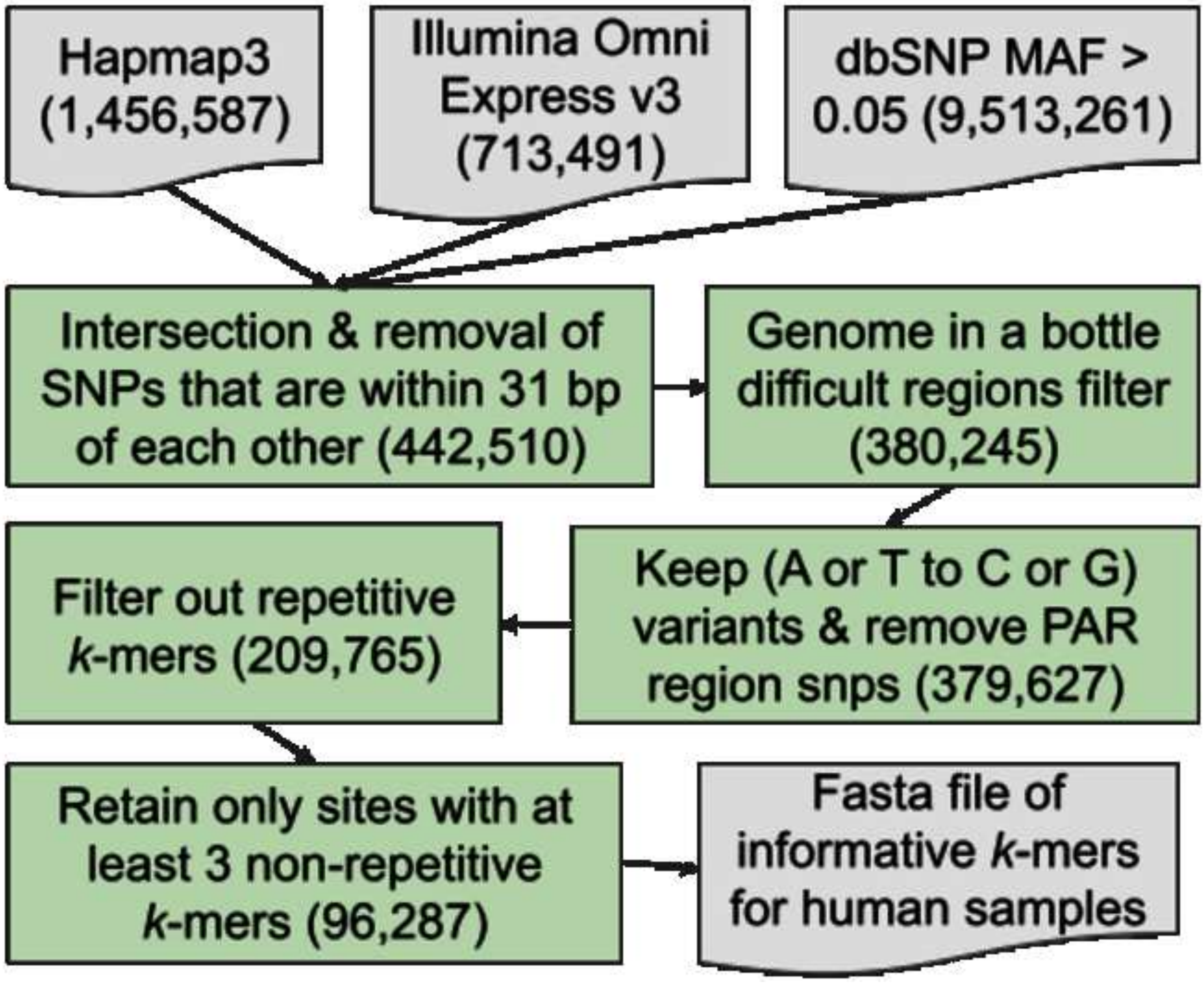

Click here to access/download;Figure;Figure2.png

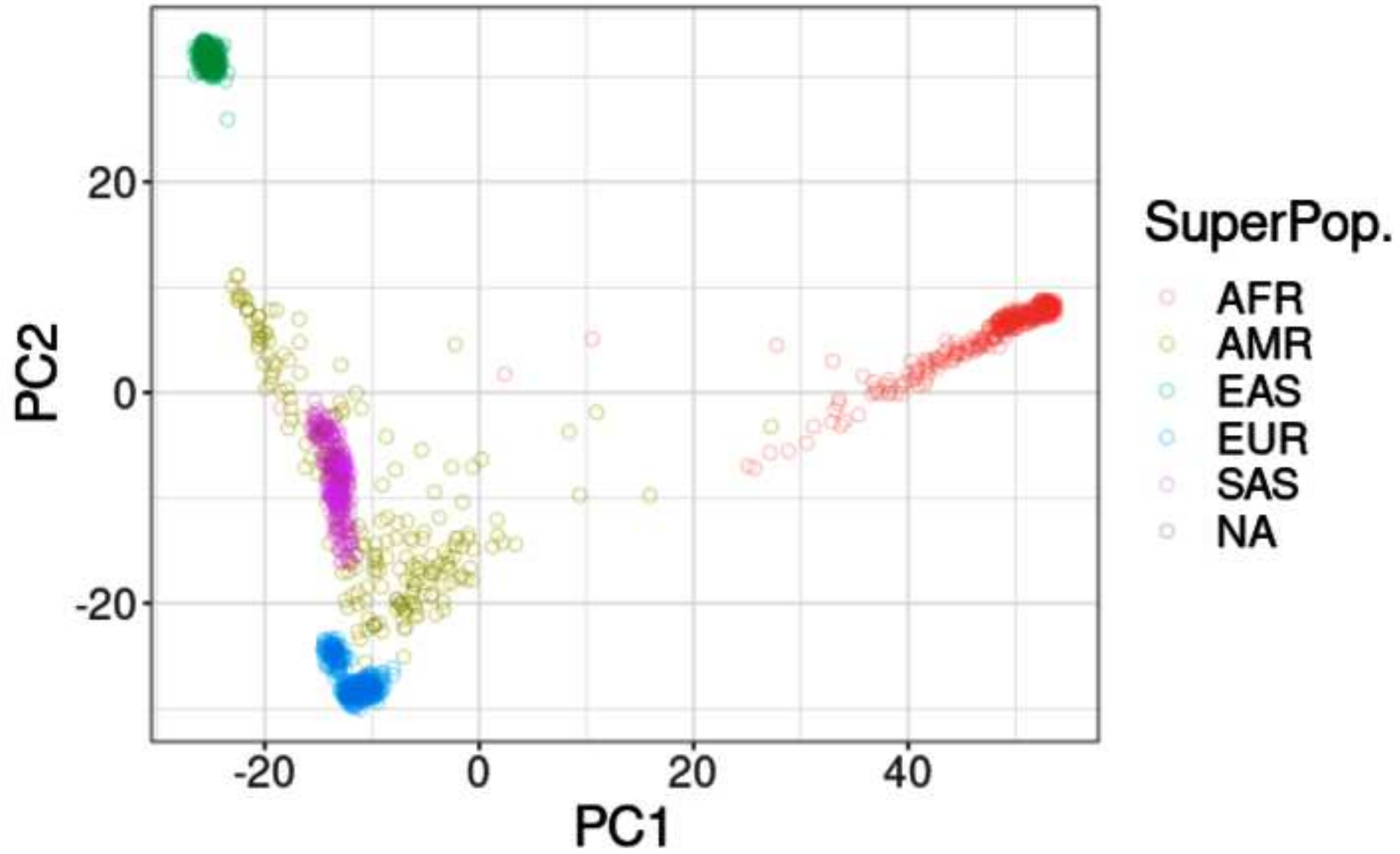

Click here to access/download;Figure;Figure3.png

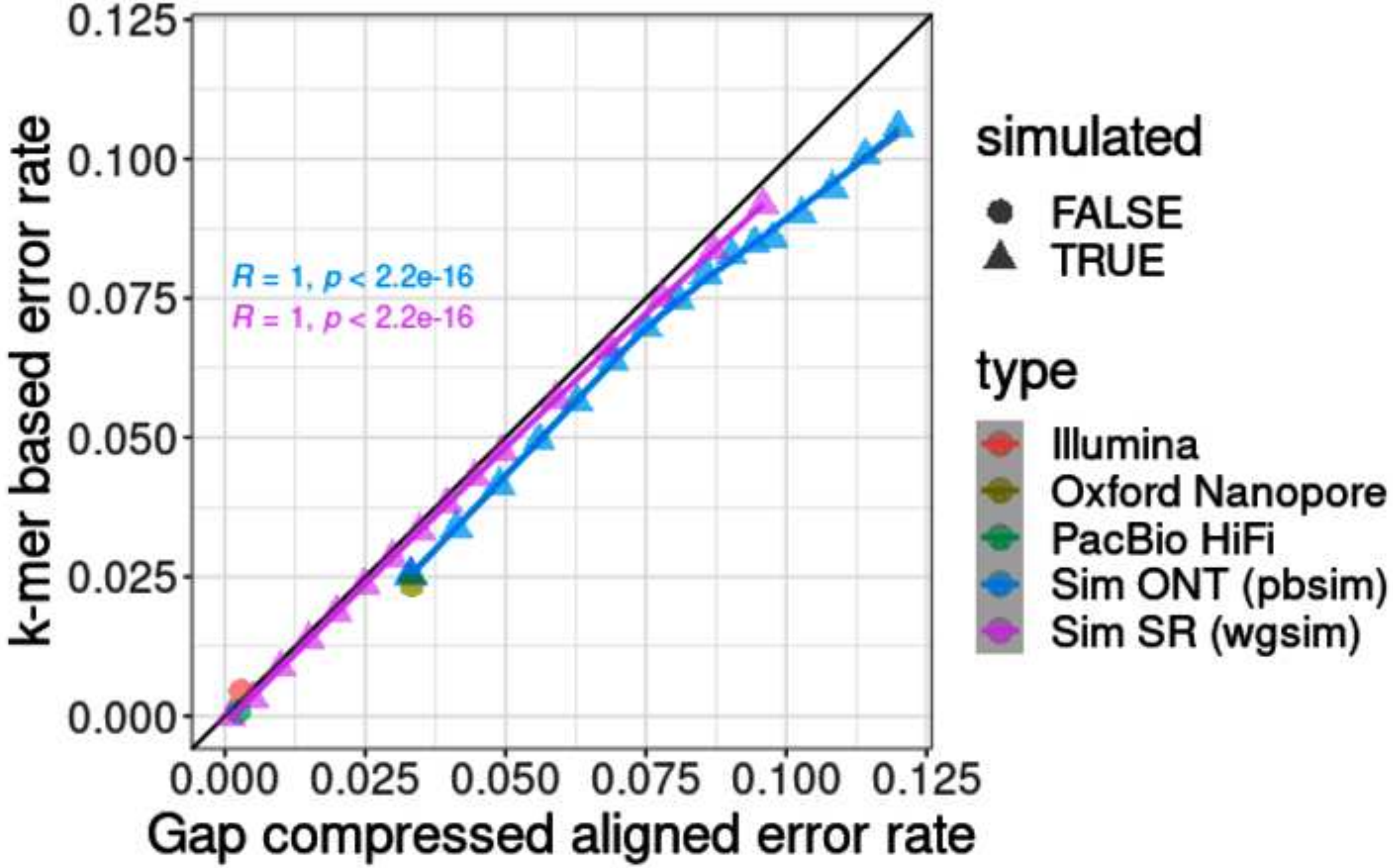

Click here to access/download;Figure;Figure4.png

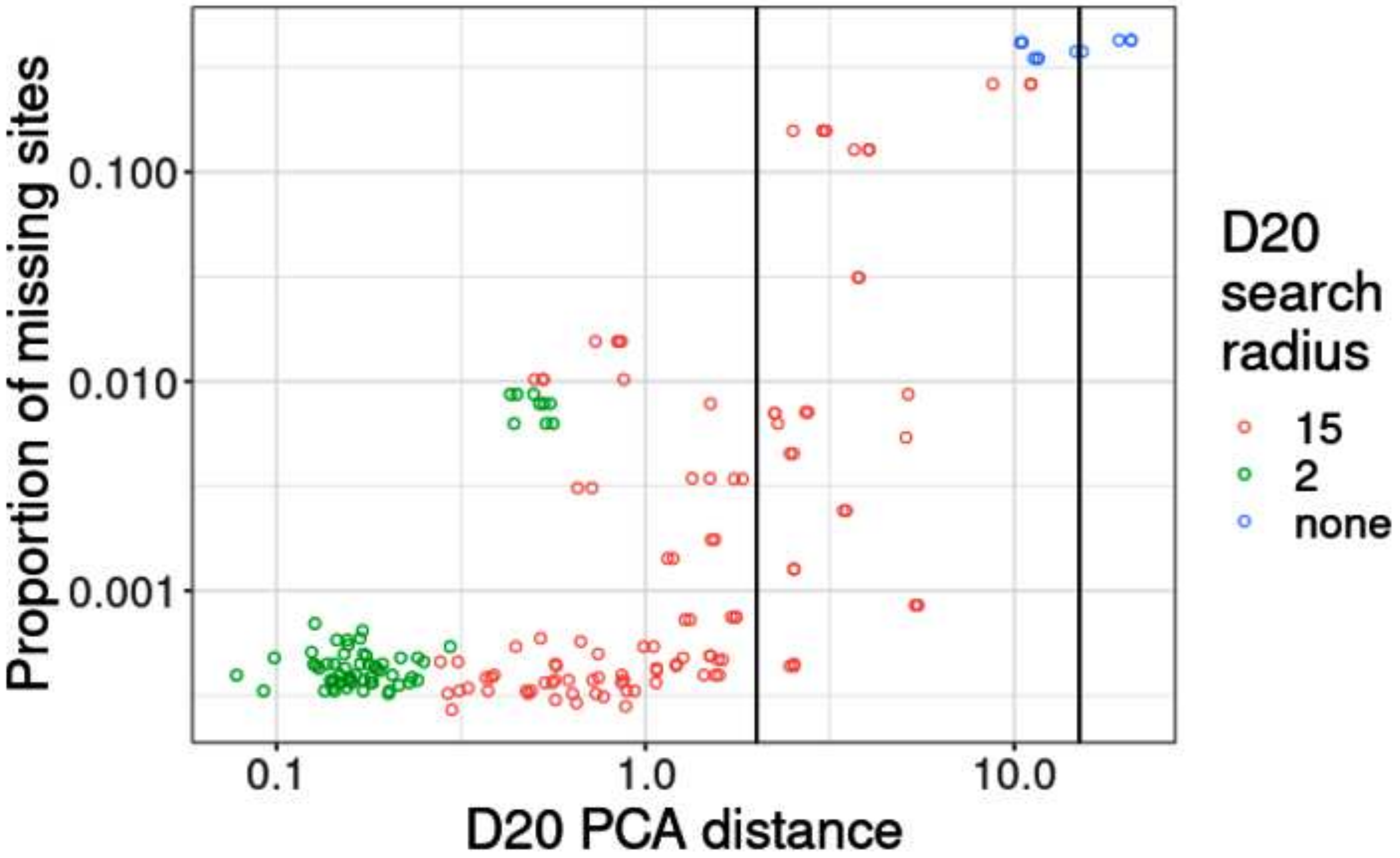

Click here to access/download;Figure;Figure5.png

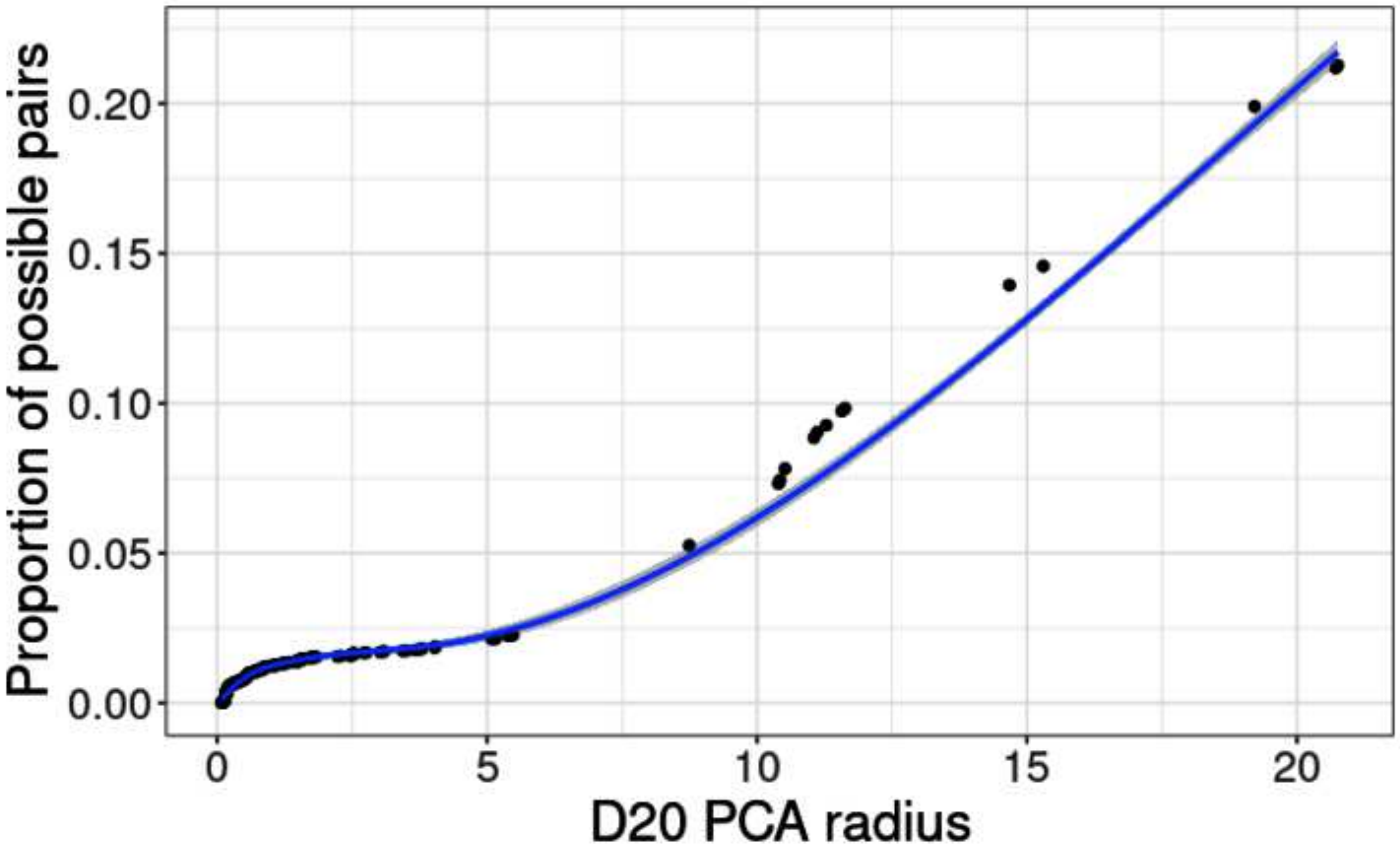

Click here to access/download;Figure;Figure6.png

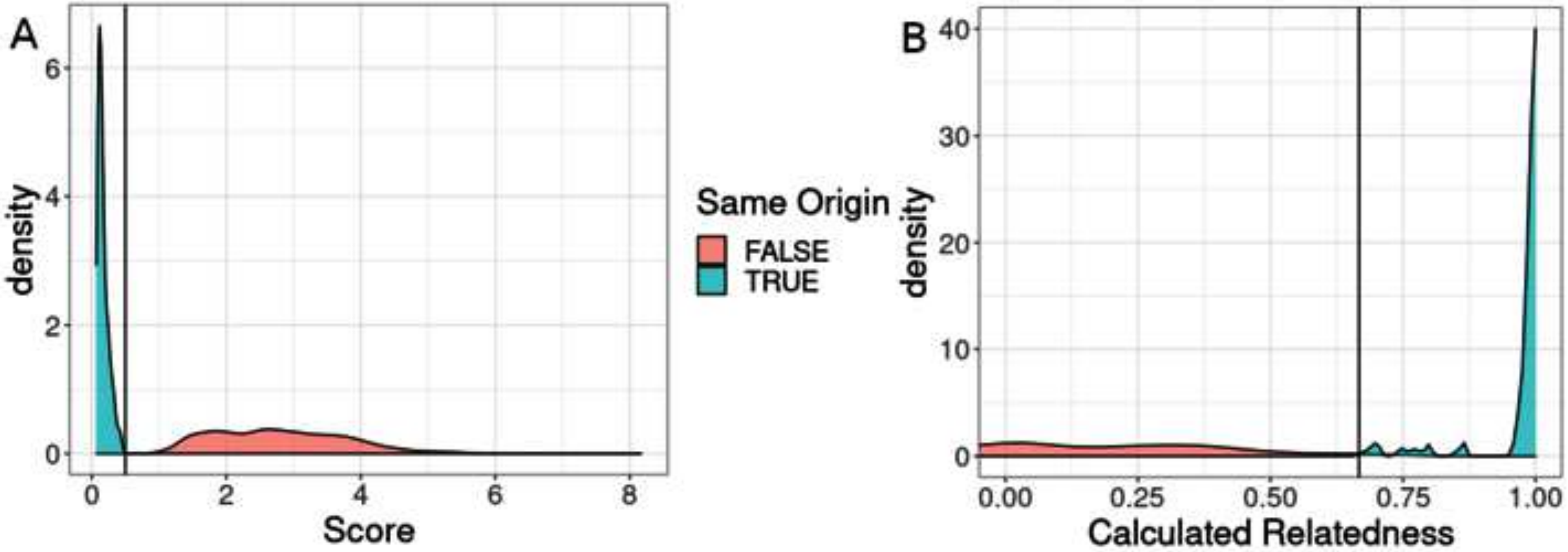

Click here to access/download;Figure;Figure7.png

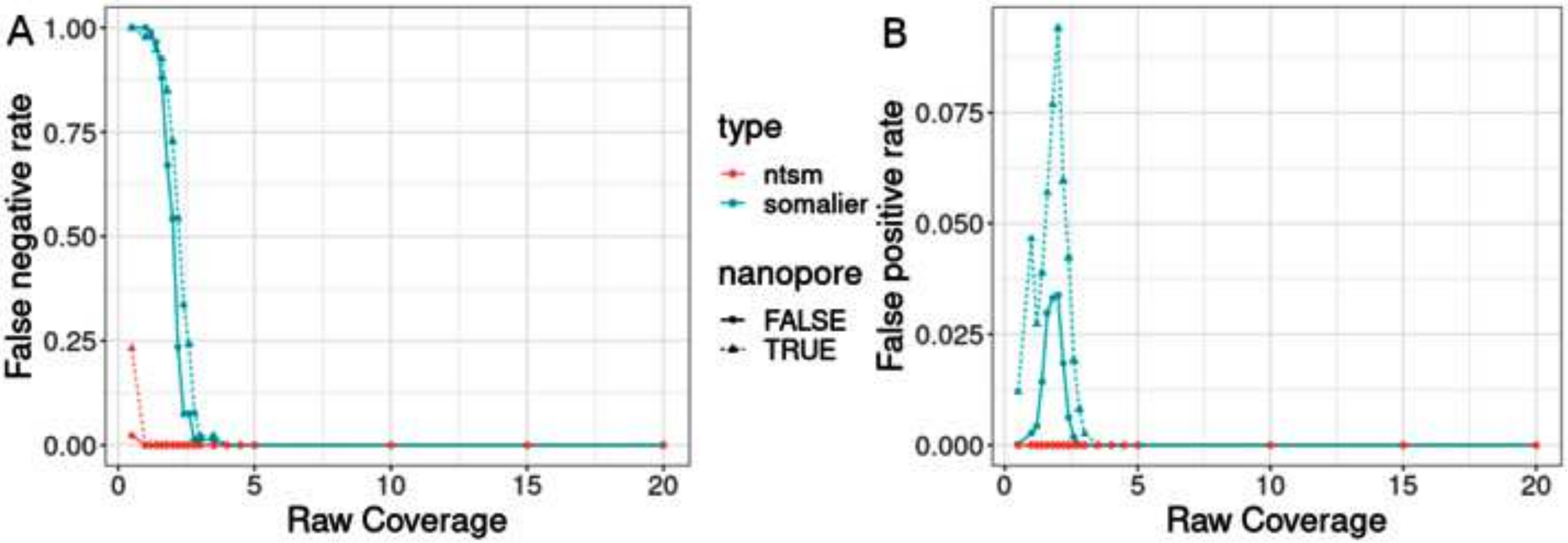

Click here to access/download;Figure;Figure8.png

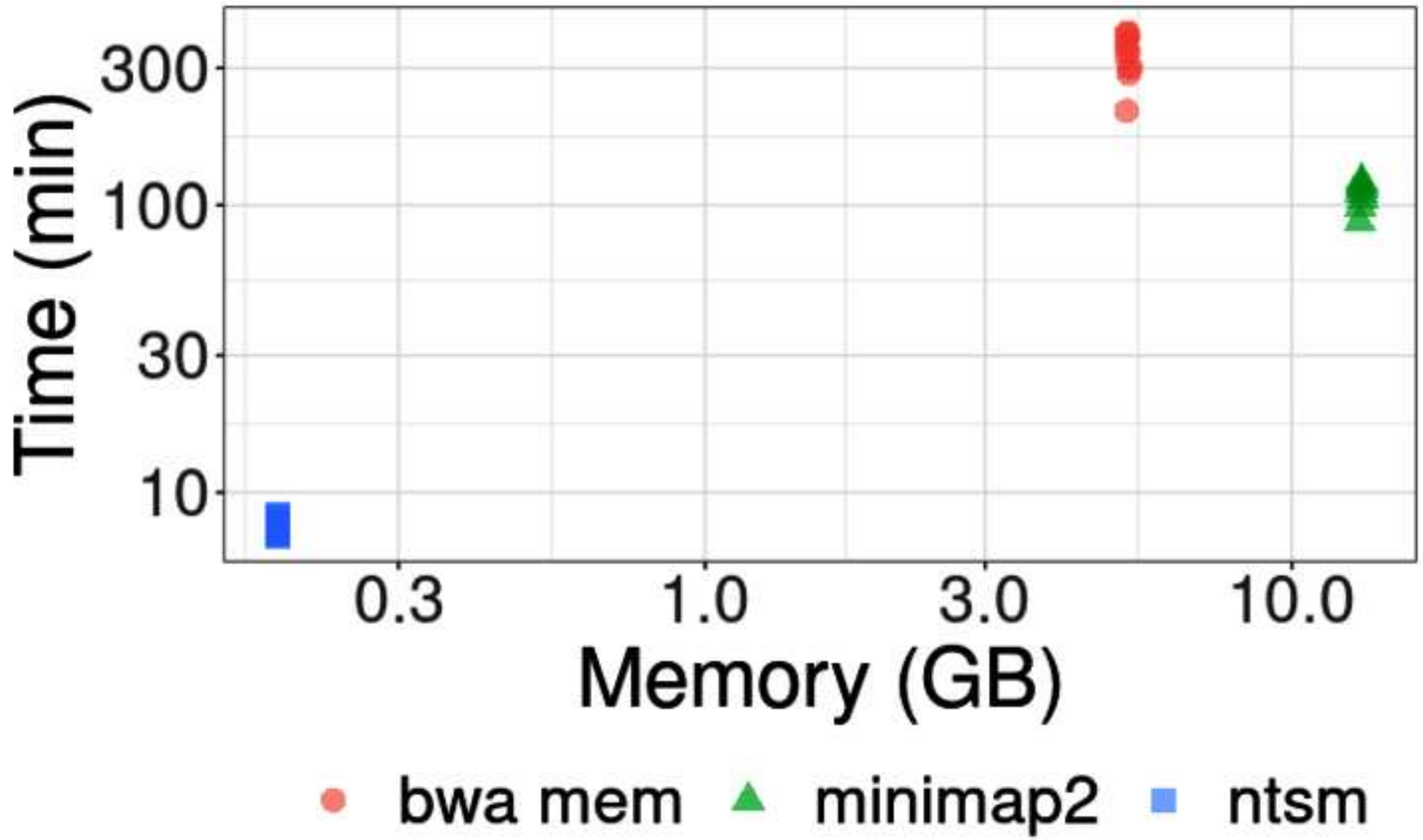

Click here to access/download;Figure;Figure9.png

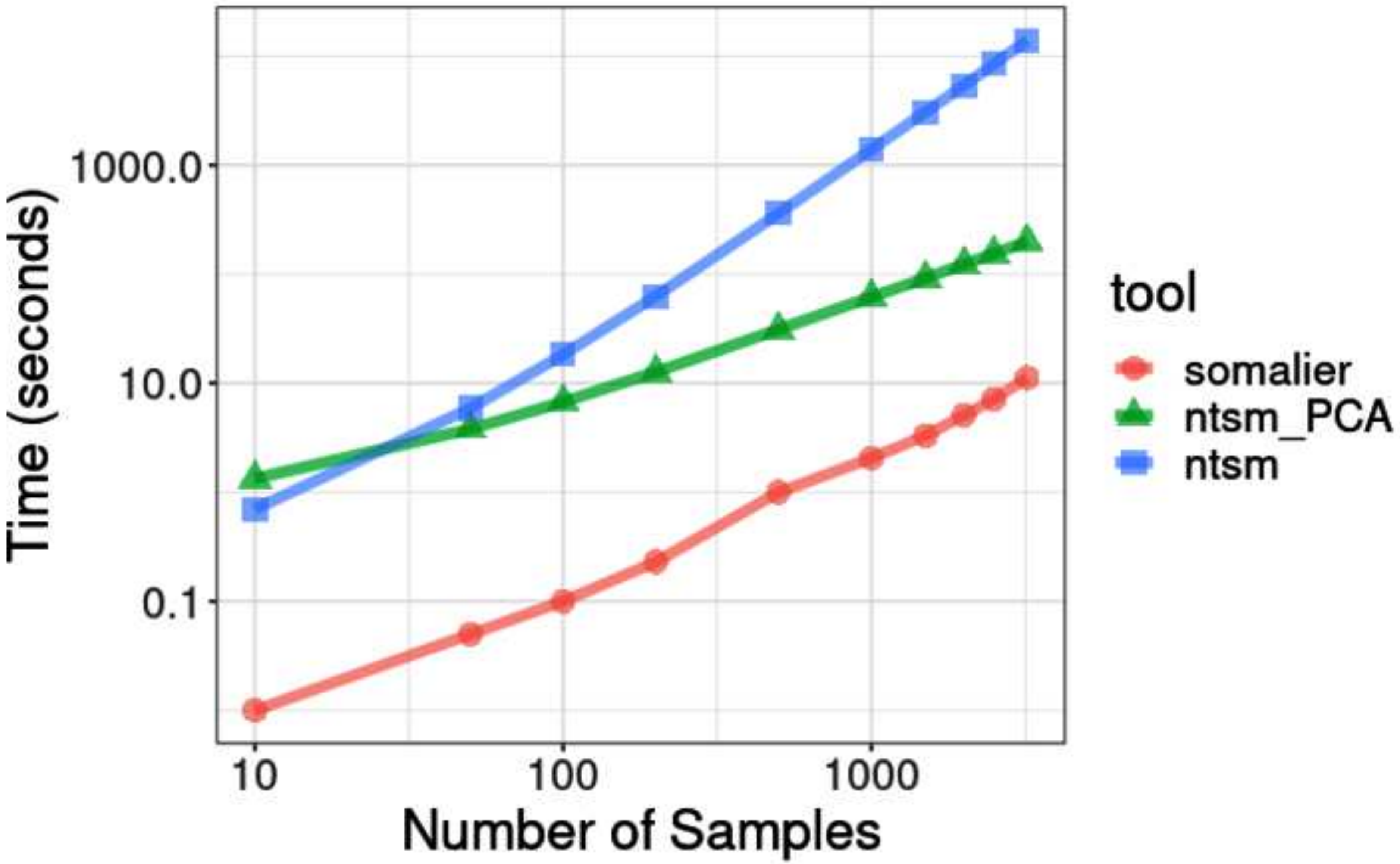

Click here to access/download;Figure;Figure10.png

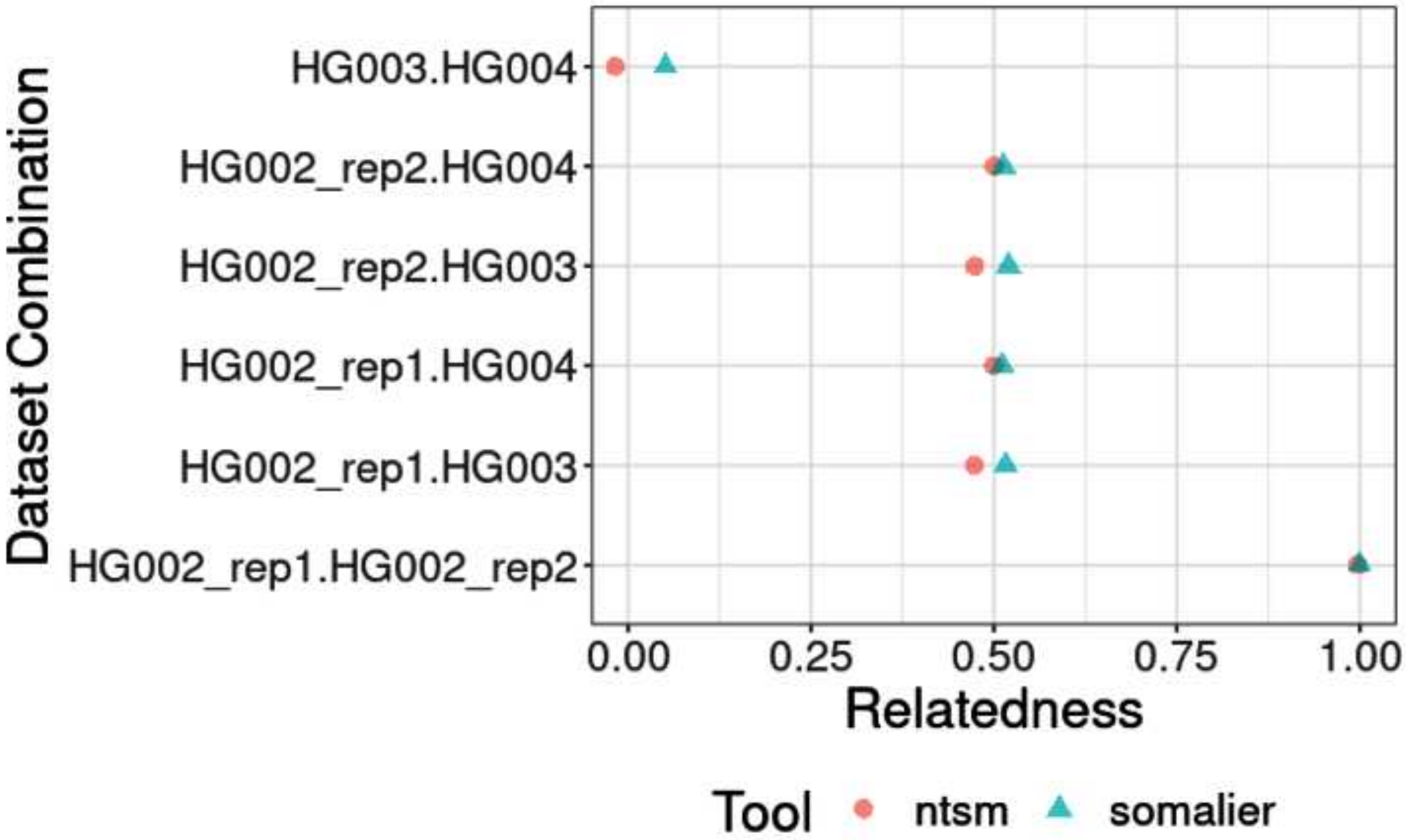

Click here to access/download;Figure;Figure11.png

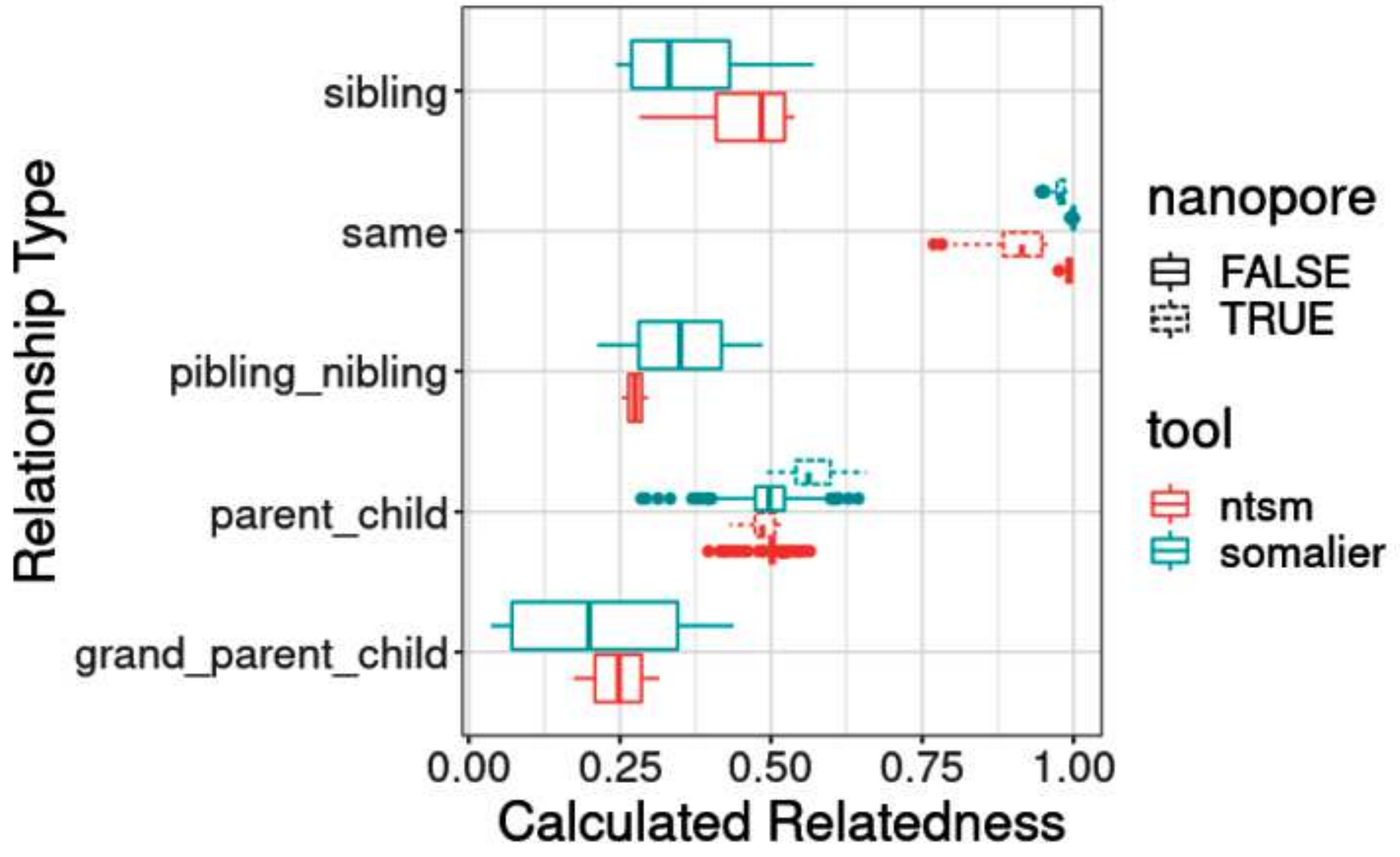

Click here to access/download
Supplementary Material
SuppFigureS1.png

Click here to access/download
Supplementary Material
SuppFigureS2.png

Click here to access/download
Supplementary Material
SuppFigureS3.png

Click here to access/download
Supplementary Material
SuppFigureS4.png

Click here to access/download
Supplementary Material
SuppFigureS5.png

Click here to access/download
Supplementary Material
SuppFigureS6.png

Click here to access/download
Supplementary Material
SuppFigureS7.png
