# Supplementary figures and images for: ntsm: an alignment-free, ultra-low-coverage, sequencing technology agnostic, intraspecies sample comparison tool for sample swap detection
Source: Gigascience. 2024 Jun 4;13:giae024. doi: 10.1093/gigascience/giae024 (PMC11148594; doi:10.1093/gigascience/giae024)

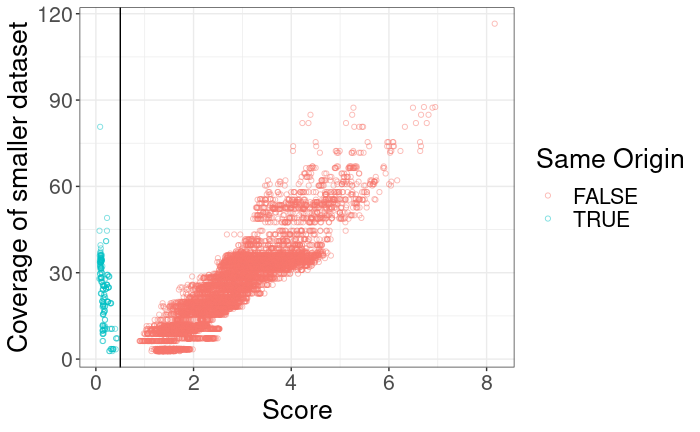

Supplement: giae024_Supplemental_Figures [file giae024_supplemental_figures.zip › SuppFigureS1.png]

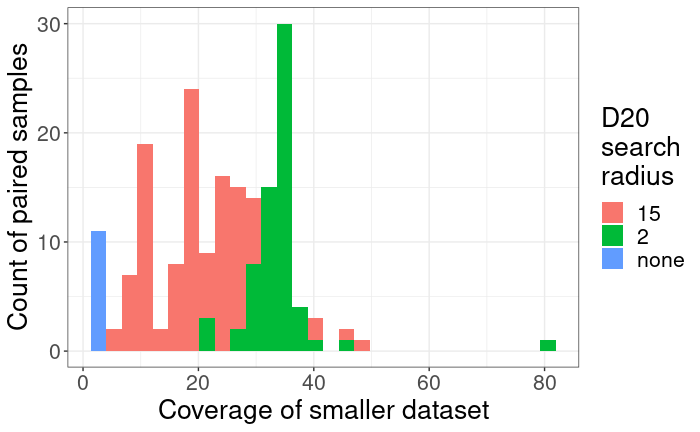

Supplement: giae024_Supplemental_Figures [file giae024_supplemental_figures.zip › SuppFigureS2.png]

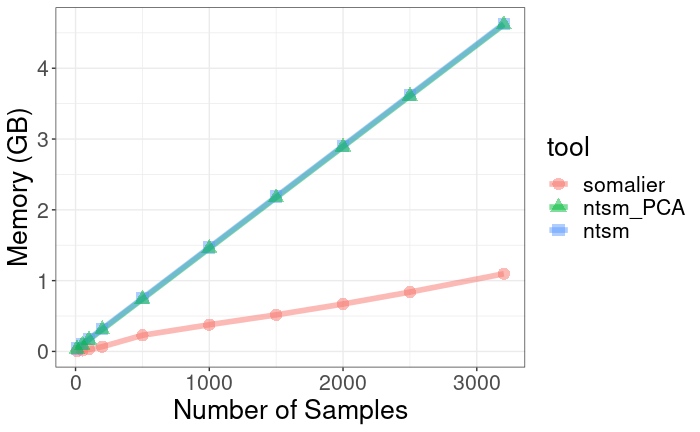

Supplement: giae024_Supplemental_Figures [file giae024_supplemental_figures.zip › SuppFigureS3.png]

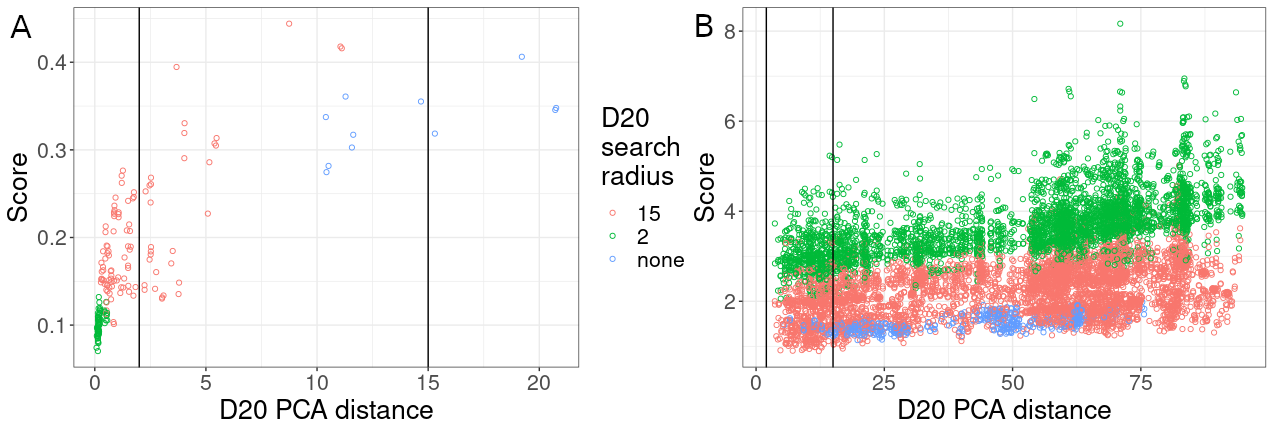

Supplement: giae024_Supplemental_Figures [file giae024_supplemental_figures.zip › SuppFigureS4.png]

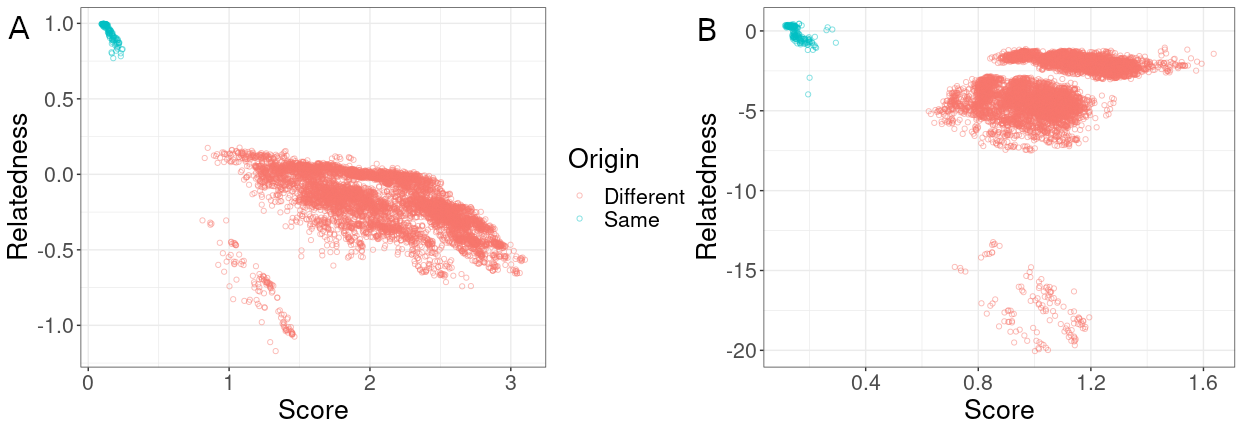

Supplement: giae024_Supplemental_Figures [file giae024_supplemental_figures.zip › SuppFigureS5.png]

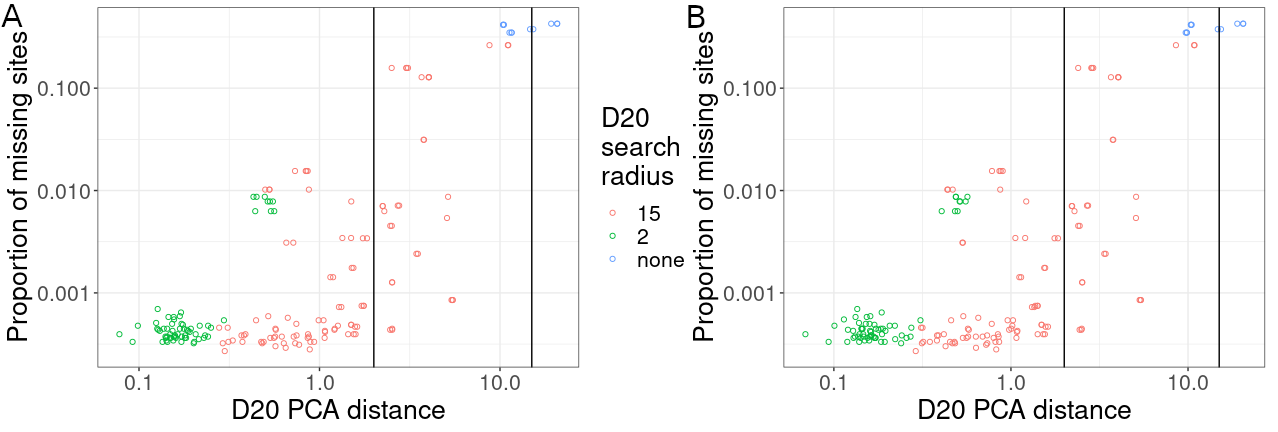

Supplement: giae024_Supplemental_Figures [file giae024_supplemental_figures.zip › SuppFigureS6.png]

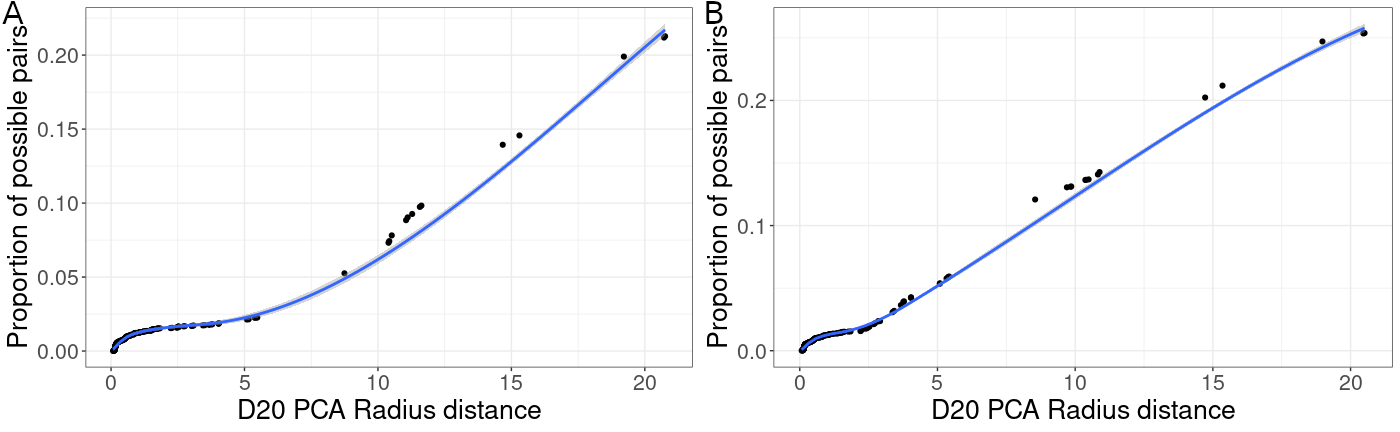

Supplement: giae024_Supplemental_Figures [file giae024_supplemental_figures.zip › SuppFigureS7.png]
