# Supplementary material for: ntsm: an alignment-free, ultra-low-coverage, sequencing technology agnostic, intraspecies sample comparison tool for sample swap detection
Source: Gigascience. 2024 Jun 4;13:giae024. doi: 10.1093/gigascience/giae024 (PMC11148594; doi:10.1093/gigascience/giae024)
Supplement: giae024_Response_to_Reviewer_Comments_Original_Submission [file giae024_response_to_reviewer_comments_original_submission.pdf]

Author's Response To Reviewer Comments

Reviewer #1:
In this manuscript, authors present a fast intra-species sample swap detecting tool, named ntsm. By counting the relevant variant k-mers from samples, it estimates the probability of each allele at sites and then uses the likelihood ratio test to detect sample swaps. Compared with the alignment-based method, Somalier, nsam performs better on low coverage data (≤5X) and is more efficient in terms of memory and computing time. The authors use PCA-based spatial index heuristic to reduce the number of sample comparisons. Of course, in my opinion, compared with the time spent on counting k-mer, the time saved by the PCA-based method is trivial. In addition, ntsm also provides other features such as error rate estimation. The tool requires population snp information, which limits its applications in practice to some extent. Overall, ntsm is a fast and practical tool for calculating intra-species sample similarity and detecting sample swaps. The writing and experiments in this paper are generally well done. There are some major and minor issues that I suggest the authors consider addressing.

Major issues:
The paper mentions that due to high error rates, nanopore data is difficult to analyze. Can the authors analyze the performance of ntsm under different error rate data? In general, alignment-based methods may perform better on high error rate data. This is very useful information for users to choose the tool.
> To be clear, we show in evaluations our method which uses counts derived from an alignment-free method performs better than Somalier which uses alignments at low coverage and high error rates (Figure 7). That being stated, we recognize the intuition of this comment as we have not made clear how this is possible and believe that we can clarify the concerns in this comment by addressing two major concepts with the manuscript:
> - Count-based similarity score: The power of our method originates from using “count information” within a joint statistical pairwise test, rather than conventional methods that collapse variant count information into individual genotyping calls that are generally weighted equally. How one acquires such count information can be done with and without alignment.
> - Alignment vs Alignment-free mapping: It is true that generally the sensitivity/specificity of alignment is generally higher than k-mer based mapping. That being said, for our specific application (counting variant coverage) we can still reach comparable performance to alignment. This is because we don’t need to map all sequences that are available, rather we only need counts from enough informative variant sites to determine their origin. In particular, we carefully select a set of variants covered by non-repetitive k-mers thus allowing for high specificity. Finally by selecting a small enough k, sensitivity issues can also be compensated for such as those caused by high error rates.
> To made sure these concepts are clear to the readers we have add the text in the “Validation and Comparison with Somalier” section in the Discussion to include: “Conceptually, our alignment-free method does not perform more accurate mapping than alignment, rather the power of our method is resultant from using count information to perform a joint statistical test that can compensate for missing data, while the alignment-free methodology enables faster analysis while maintaining adequate performance compared to alignment. Specifically, our unique application of sample swap detection lends itself to being able to select variant sites which do not contain repetitive k-mers (ensure high specificity) as only a subset of all informative variants are needed for this type of analysis. Finally, to maintain a high sensitivity, relatively small (19-mers) are used and can still detect useful k-mers in high error rate data.”

The authors use the PCA-based spatial index heuristic to reduce the number of pairwise comparisons. However, the relation between PCA distarnce and similarity score is not clear here. How to ensure that samples with similarity scores less than the threshold are within the search radius?
> We have included Supplementary Figure S4, showing how the PCA distance relates to our score. At least in samples that match, it shows score and PCA distance are correlated though is far more variable if the samples do not match. If robust thresholds for the PCA distance radius are selected in most cases the PCA-based heuristic will not decrease the sensitivity of our method but in some cases this may reduce sensitivity. As our lowest threshold is unbounded (determined by a high error rate and large number of missing sites), it would require exceptional circumstances which we did not observe in our tests. While exploring how the use of our PCA heuristic affects the performance of our algorithm we have also changed

figure 5 to more clearly show the heuristic reduces the number of total comparisons that would hypothetically be considered as different PCA distance radii are used in the search.

The paper involves two metrics, say, similarity score and relatedness, to detect sample swaps. Can the authors analyze the relation between them to help readers understand the advantages and disadvantages of the two methods?
> We chose to use similarity score over relatedness because it resolves if samples are of the same origin better, especially when data is low in coverage (thus more missing sites). We have included Supplementary Figure S5, showing that though relatedness may be a decent metric at 20x, it begins to fail at 5x raw coverage, while score remains a good metric to separate the matching and non-matching pairs.

Minor issues:
In the "Conlusions" section, the second "useful" in the sentence "this method provides other useful information useful in QC" is redundant.
> Reworded as “this method also provides information useful in QC”

"R=1, p<2.2e-16" in Figure 3 is not explained.
> We include “The Pearson correlation coefficient (R) is provided showing high correlation between k-mer based error rate estimate of the true error rate.”

In the "Sequencing error rate estimation" section, the variable n is not explained.
> In that section we had written “To relate these values to each other, we need an expected number of k-mers n assuming no error”, to make it clearer we have written: “To relate these values to each other, we need to know when no error is present, the expected number of k-mers n”

In Figure 9, the case of the first letter of two y-axis labels (time) is inconsistent.
> We have replaced the plot for clarity (see reviewer 2’s comments) and have included the unit “seconds”.

Reviewer #2:
In this paper, the authors have presented a tool, ntsm, which utilizes the k-mer distribution information directly from raw sequencing data for sample swap detection. The approach of bypassing the reference genome alignment step and saving computational resources is commendable. Utilizing k-mers for reference-free and de novo analysis of sequencing data is a valuable application. The authors have demonstrated the impressive performance of ntsm on low coverage data through experimental results presented in the manuscript, showcasing its strengths in terms of sensitivity, accuracy. However, while ntsm eliminates the need for reference genome alignment, it still relies on a pre-defined set of variant sites and pre-built PCA rotation matrices. This raises doubts about the true reference-free nature of ntsm and raises concerns about its generalizability to other species.

Major comments:
The concept of reference-free: I believe that ntsm's approach is not truly reference-free. In order to use ntsm, it requires the use of existing high-quality population SNP sites and kmers from the human reference genome. Additionally, the population PCA results are used to assist in pairwise comparisons between samples. Both of these information can only be obtained when a reference genome is available. A true reference-free tool would be applicable to species without a reference genome, such as SPLASH (Chaung et al., 2023, Cell). ntsm can be considered as an alignment-free or kmer-based tool.
> Our method is indeed not reference-free and we hope that this did not come across as the case within the manuscript. We have made this more explicit in the Discussion section re-writing: “We recognize that a tool that uses reference-free information like raw k-mer spectrum information would be more desirable than our reference-based method using k-mers from a set of reference-derived sites”. It is not our intention to imply we are using a reference-free method and it would be appreciated if the reviewer could point out any additional sections of text where it seems like we imply this.

The reduction of computational costs: NTSM differs from Somalier in its computational workflow. To compare the computational costs or time, a holistic end-to-end comparison is necessary, rather than timing individual steps such as kmer counting and sample pairwise comparison separately. Conducting an end-to-end comparison for an analysis task allows users to have a comprehensive understanding of the tool's time

and cost consumption. Furthermore, when comparing software, it is important to allocate computational resources fairly. For example, ntsm utilizes 16 threads in the 'Sample comparison process' stage, while for the 'k-mer counting (ntsm) vs. alignment (somalier)' stage, tools like bwa and minimap2, which can utilize multiple threads, were run using a single thread.
> We have clarified the workflow wIthin this section by including the text: “Workflow for processing raw read data for Somalier and ntsm can be thought of in 2 major steps: (1) Per sample read processing to obtain variant site information and (2) Joint sample variant site information all-to-all comparisons to obtain similarity. In Somalier, step 1 entails taking raw reads, aligning them to a reference (using aligners like bwa or minimap2, depending on the read type), sorting and indexing them (using samtools), and finally extracting the variant information for specific sites into a bitvector (somalier extract). The computational resources required for this step are dominated by the alignment stage. Finally, step 2 takes each extracted bitvector and runs pairwise comparison between all of them to obtain relatedness. While in ntsm, step 1 entails taking raw reads and counting k-mers for specific sites (ntsmCount). These count files are then compared (ntsmEval) using the count information directly in step 2.”
> We agree an end to end comparison would be ideal. However, the two steps mentioned above cannot be coupled in a meaningful way in most practical situations it will be dominated by the per sample alignment. We show the cost of the second stage as due diligence towards a process that scales quadratically and though we know is more sensitive it comes at the cost of computational speed, even if a minor component of the overall workflow computational cost. We agree that computational results can be misleading in the way we have previously presented them and reverted all tests in this section to use a single thread.
> We have replaced figure 9 with another plot with results only using a single thread. So comparisons can be seen more clearly even with large differences, the new plot we have changed the axes to be shown in log scale.

Sensitivity and Specificity: More experimental details are needed. In the section 'Sensitivity and Specificity of Sample Swaps,' were the results obtained using the 39 HPRC samples? Did it include their Hi-C data? For Fig 6, did the results come from all sequencing datasets of the 39 samples, including Illumina and ONT? Since the results was obtained using full coverage, would the threshold change at lower coverage? For Fig 7, which demonstrates ntsm's results, was PCA information used as an auxiliary? Does the use of PCA information impact Sensitivity and Specificity?
> We have re-rewritten the section to include: “To determine a threshold, we ran the full coverage of all the HPRC sample datasets (including all available data types) mentioned above for Somalier and manually picked a threshold that provided good separations between samples with the same origins from those with different origins (Figure 6)”
> In the manuscript we noted that “Somalier does not provide an automated means of determining what should be considered similar”, so had to pick something reasonable from their output to do proper comparisons. Relatedness between two samples has a ground truth state and thus as a metric should in theory not be affected by coverage. Because Somalier can only merely estimate relatedness, it could be argued that a reasonable threshold is indeed affected by low coverage. It depends if one values specificity or sensitivity, though we feel we picked a reasonable one, considering we show both a higher sensitivity and specificity using ntsm (rather than just showing a higher sensitivity or specificity).
> The PCA information is used in Figure 7 which we also observe results in that figure in the same results with and without our PCA heuristic in this case and we now mention this is the Figure 7 caption. We note however that in theory the PCA heuristic could impact sensitivity though is unlikely for more details see our response to Reviewer 1’s major comment #2.

Regarding PCA-based method: The 39 HPRC samples used in the study are actually part of the 3,202 samples from the 1000 Genomes Project. Therefore, it is important to clarify whether the PCA matrix used in the study already includes information from these 39 samples. From a rigorous experimental design perspective, a precomputed PCA matrix should not include information from the 39 samples. Otherwise, the effect of the PCA matrix on these 39 samples may be overestimated. It raises questions about whether the same results can be achieved on non-1000 Genomes Project samples.
> Though somewhat different this comment touches on a concept similar to using data found in a training dataset in your test dataset. We agree with the reviewers here that under ideal circumstances our HPRC dataset would be completely independent of the 1000 genomes VCF file used to create our PCA. With this in mind we have re-performed our analysis using a PCA derived from 1000 genome samples minus any sample IDs found in the HPRC dataset. We have recreated relevant figures to show the effects of such a

change (Supplementary figure S6,S7). In addition we confirmed that the results for figure 7 remain the same even with this change. We hope that this shows adequately that the addition of these sample IDs in our PCA does not significantly change the major findings of using our heuristic the paper.

The applicability of the tool: In order to expand the applicability of ntsm to a wider range of species, two aspects need to be addressed: Provide detailed information on customizing the sites file.
1. From the site files available in ntsm code repository on GitHub, the process of selecting variant sites seems to be more complex than what is described in the manuscript, involving more than just SNP variants.
> From an input VCF file and reference genome, we have added to the github to a script and how to use it in the Readme. It includes a script to generate a PCA if given a multiVCF file for the same genome if the user wishes to use the heuristic.

2. The sites and PCA files should be user-customizable inputs instead of being built-in. This limitation restricts the application of ntsm to other species.
> The PCA inputs are not built-in, though the error rate calculations assume that a human genome. To adjust, change the parameter (-g) in ntsmEval to the rough expected size of the genome (diploid size). This is also reflected in the readme of the Github repository.

Minor comments:
The manuscript appears to have been hastily written and requires further polish by the authors.
In Figure 6, A and B seem to be labeled incorrectly.
> We have fixed the caption to reflect the correct labels.

In Figure 9, the two subplots have different y-axes, one labeled "min" and the other labeled "s." Could you clarify what each subplot is illustrating?
> Reviewer 1 also had the same issues with the plot, a sign that it is confusing in general. We have replaced the 2 plots with a single plot using log scales on both axes. We have updated the figure to show “seconds” so hopefully this is more clear.

When mentioning HPRC for the first time, it would be helpful to provide the full name and explanation of the acronym. However, the full explanation appears in the next paragraph.
> In the original version, we actually mention the HPRC for the first time in the introduction, which is where the full name is written but then also again write the full name again later on the third time it is mentioned (where Reviewer 2 is mentioning). To maintain consistency with correct convention we have removed the full name from the third time it is mentioned.

"We then keep only purine to pyrimidine (A or T to G or C) variants, as final insurance against possible human error influencing this tool" It seems there may be a mistake or confusion in the sentence. The writer should indeed mention "A/G <-> C/T" instead of "A/T <-> G/C" to accurately describe purine to pyrimidine variants. The writer may have made an error in describing the nucleotide exchange, or it could be a typographical mistake.
> Yes this is an oversight, it indeed has nothing to do with purine and pyrimidine status. We have rewritten this section to “We then keep only (A or T to G or C) variants, as human-error induced DNA strand mix ups are common but less likely when non-complementary base polymorphism are used.” We have also fixed figure 1 to reflect this fix. We are grateful to reviewer 2 for noticing this.

There is a typo in the formula for estimating sequencing error rate. (nm)·log(1-… …
> Thank you, we have corrected the formula to “(n-n)·log(1-… …”
