# Supplementary material for: ntsm: an alignment-free, ultra-low-coverage, sequencing technology agnostic, intraspecies sample comparison tool for sample swap detection
Source: Gigascience. 2024 Jun 4;13:giae024. doi: 10.1093/gigascience/giae024 (PMC11148594; doi:10.1093/gigascience/giae024)
Supplement: giae024_Response_to_Reviewer_Comments_Revision_1 [file giae024_response_to_reviewer_comments_revision_1.pdf]

Author's Response To Reviewer Comments

Reviewer #1: In the revised manuscript, the authors addressed the issues I raised by conducting additional work. They presented more results to demonstrate that similarity score is a better metric for detecting sample swaps and the PCA-based screening method is effective. While Figure 7 shows the results for the Nanopore datasets, the error rates of these are not given. If possible, I hope the authors can provide the error rates of benchmark datesets. Here are some other issues.
1. The format of references is messy. It need further examination and correction.
> We are following the Gigascience citation format using a citation manager. We have rechecked the citations and found that the autoformatting did not execute correctly and have fixed the problems we found.
2. The sentence "To observe the computational expense of this process we took subsamples of the 1000 Genomes VCF files and measured time and memory used to compute relatedness between the samples. " in section "Sample comparison process" may be incorrect. "compute relatedness" should be "detect sample swaps". I think ntsm_PCA cannot be used to compute relatedness.
> We have rewritten the text to: "To observe the computational expense of this process we created simulated counts files (for ntsm) and bit vector files (for Somalier) for the samples found in the 1000 Genomes VCF and measured time and memory used to detect sample swaps between subsamples at different sizes"
3. Running time and memory usage of ntsm for computing relatedness should be given.
> Relatedness computation is included in the output of all sample pairs reported in our standard output. We have included this sentence in the k-mer relatedness section: "Relatedness computation is included for all sample pairs reported"
4. In Figure 3, the sentence "The Pearson correlation coefficient (R) is provided showing high correlation between k-mer based error rate estimate of the true error rate" is grammatically incorrect. "PCA-based screen approach" should be "PCA-based screening approach". "methods" in "To test our relatedness estimation methods" should be "method".
> Changed to "The Pearson correlation coefficient (R) is provided showing high correlation between our k-mer based error rate estimate to the gap compressed error rate computed from alignments"
> "PCA-based screen approach" changed to "PCA-based screening approach"
> "relatedness estimation methods" changed to "relatedness estimation method"

Reviewer #2: I thank authors for taking time and giving detailed response to my comments, which have resolved most of my major concerns in the previous round. I however still have a few comments related to the time comparisons.

The author clearly divides the workflow of Somalier and ntsm into step 1 and step 2. This is reasonable. However, the comparisons lack a presentation of the total time consumption for step 1 and step 2. As a reader, what I see is that Figure 8 shows that in step 1, ntsm is much faster than Somalier, while Figure 9 shows that in step 2, ntsm is much slower than Somalier, especially when considering that Figure 9 uses log scale on the axes, indicating a significant difference between ntsm and Somalier in step 2. This does not allow the reader to quickly reach the conclusion stated in the abstract, "this tool (ntsm) is faster than alignment, it can save an immense degree of computational resources." I strongly recommend that the author use a table to present the time consumption for step 1, step 2, and the overall workflow for sample quantities such as 10, 100, 1000, 3000, etc.

> We acknowledge that we do not make it clear to read that conclusion in the manuscript as it should be in the manuscript and have made the following revisions for address this flaw:
> We expanded and revised the "Validation and Comparison with Somalier" in the discussion section. The full added text is we believe is should alleviate these misconceptions most significant section relating to reviewer 2 concerns are as follows: "the pairwise evaluation times for ntsm on a single thread were 4 hours for ntsm, and ~3 mins for ntsm with our PCA heuristic and 11 seconds for Somalier. For perspective, if we had used raw illumina short reads rather than a VCF file, 3202 samples would take at over 5 CPU years to align with bwa mem as compared to k-mer counting in only 43 CPU days"

> Raw data for replication for figure 9 is included with our submission to Gigascience, however we have also included the results in a table as Supplementary table S2.

Additionally, are the results in Figure 8 based on a single sample or on all samples from Table S1?
> It is based on multiple datasets from Table S1. Some of these dataset did not have sufficient coverage for this test or were added to the manuscript after additional dataset included, though we believe we have a large enough set of 2x reads (39 datasets) to derive the conclusions regarding performance. As part of our submission to Gigascience, we have included the raw tsv for generating figure 8 listing which datasets were used along with the time and memory used.
Can ntsm use multiple threads in the k-mer counting step?
By using multiple threads in the k-mer counting step, it further reduces the time consumption of step 1 and enhances the practical applicability of ntsm in large cohort studies. After all, alignment is almost an unavoidable step in low-coverage whole-genome re-sequencing projects, while k-mer counting is not. Only when the k-mer counting step is fast enough, performing k-mer counting for sample swap detection will not add significant computational burden to the entire project.
> Yes indeed ntsm can use multiple threads at all stages. In the implementation details section on k-mer counts we already state: "Multiple files and threads can be used on the same instance of the counting tool for speed, or run separately on different files and later merged". We largely chose not to use multiple threads in our testing to keep evaluation simple as I/O would begin limiting the process. Additionally we state: "The counting can be set to terminate early if sufficient k-mer coverage is obtained" as a complete k-mer count isn't needed to get enough counts for accurate sample swap prediction further reducing runtime.

Please consider the following suggestions for the descriptions of results:
1. "Somalier, utilizing a bitvector based comparison method is much more optimized for speed than our count based method, however we show that our PCA-based screening method is still competitive."
This conclusion was derived when ntsm was using 16 threads. However, in the new Figure 9, the speed of the PCA-based screening method is significantly slower than Somalier, when both ntsm and Somalier are using a single thread.
> We have revised the text in this section as follows: "Somalier, utilizing a bitvector based comparison method is much more optimized for speed than our count based method. However for the sample comparison stage ntsm is capable of using more than one thread which can help close the gap in speed. When using 16 threads with ntsm in PCA mode we were able to remain competitive at 32 seconds compared to 15 seconds respectively on 3202 samples. For sample comparison, Somalier is currently unable to use multiple threads."

2. "Unlike Somalier, ntsm is capable of using more than one thread which can help close the gap in speed."
The author points out that ntsm can alleviate the speed disadvantage in step 2 by using multiple threads. However, the author overlooks the fact that Somalier can also mitigate the speed disadvantage in step 1 by utilizing the multi-threading capabilities of tools like BWA and Minimap2. To ensure fairness in the description, it is suggested to remove this statement.
> We are only qualifying this statement in the sample comparison process (i.e. step 2), though to reduce confusion we have added the qualifier: "For sample comparison" in this section in addition to the textual changes based on the previous above. Overall, we do not believe that we are unfairly misrepresenting how multithreading capabilities influence performance as ntsm k-mer counting is also capable of using multiple threads like BWA and Minimap2 in step 1.

Typos:
1. In Response to Reviewers " we have corrected the formula to (n-n)·log(1-…", it should be "n-m".
> We apologize for this oversight and thank the reviewer for their thoroughness. We cannot retroactively change this in the reviewer response but have ensured it was indeed corrected correctly in the last revision in text manuscript (i.e. "n-m").
2. "Though most of the time reads will be used as input, Both Somalier and ntsm" , it should be "both".
> Thank you, this has been fixed in the manuscript.
