# Supplementary material for: ntsm: an alignment-free, ultra-low-coverage, sequencing technology agnostic, intraspecies sample comparison tool for sample swap detection
Source: Gigascience. 2024 Jun 4;13:giae024. doi: 10.1093/gigascience/giae024 (PMC11148594; doi:10.1093/gigascience/giae024)
Supplement: giae024_Response_to_Reviewer_Comments_Revision_2 [file giae024_response_to_reviewer_comments_revision_2.pdf]

Author's Response To Reviewer Comments

Editor suggested changes made. Please let me know if there are any issues.
