# Supplementary material for: ntsm: an alignment-free, ultra-low-coverage, sequencing technology agnostic, intraspecies sample comparison tool for sample swap detection
Source: Gigascience. 2024 Jun 4;13:giae024. doi: 10.1093/gigascience/giae024 (PMC11148594; doi:10.1093/gigascience/giae024)
Supplement: giae024_Reviewer_1_Report_Original_Submission — Jianxin Wang -- 12/26/2023 Reviewed [file giae024_reviewer_1_report_original_submission.pdf]

Reviewer Report

Title: ntsm: an alignment-free, ultra low coverage, sequencing technology agnostic, intraspecies sample comparison tool for sample swap detection

Version: Original Submission **Date:** 12/26/2023

Reviewer name: Jianxin Wang

Reviewer Comments to Author:

In this manuscript, authors present a fast intra-species sample swap detecting tool, named ntsm. By counting the relevant variant k-mers from samples, it estimates the probability of each allele at sites and then uses the likelihood ratio test to detect sample swaps. Compared with the alignment-based method, Somalier, nsam performs better on low coverage data (â‰¤5X) and is more efficient in terms of memory and computing time. The authors use PCA-based spatial index heuristic to reduce the number of sample comparisons. Of course, in my opinion, compared with the time spent on counting k-mer, the time saved by the PCA-based method is trivial. In addition, ntsm also provides other features such as error rate estimation. The tool requires population snp information, which limits its applications in practice to some extent. Overall, ntsm is a fast and practical tool for calculating intra-species sample similarity and detecting sample swaps. The writing and experiments in this paper are generally well done. There are some major and minor issues that I suggest the authors consider addressing.Major issues:The paper mentions that due to high error rates, nanopore data is difficult to analyze. Can the authors analyze the performance of ntsm under different error rate data? In general, alignment-based methods may perform better on high error rate data. This is very useful information for users to choose the tool.The authors use the PCA-based spatial index heuristic to reduce the number of pairwise comparisons. However, the relation between PCA distance and similarity score is not clear here. How to ensure that samples with similarity scores less than the threshold are within the search radius?The paper involves two metrics, say, similarity score and relatedness, to detect sample swaps. Can the authors analyze the relation between them to help readers understand the advantages and disadvantages of the two methods?Minor issues:In the "Conlusions" section, the second "useful" in the sentence "this method provides other useful information useful in QC" is redundant."R=1, p<2.2e-16" in Figure 3 is not explained.In the "Sequencing error rate estimation" section, the variable n is not explained.In Figure 9, the case of the first letter of two y-axis labels (time) is inconsistent.

Level of Interest

Please indicate how interesting you found the manuscript: Choose an item.

Quality of Written English

Please indicate the quality of language in the manuscript: Choose an item.

Declaration of Competing Interests

Please complete a declaration of competing interests, considering the following questions:

- Have you in the past five years received reimbursements, fees, funding, or salary from an organisation that may in any way gain or lose financially from the publication of this manuscript, either now or in the future?
- Do you hold any stocks or shares in an organisation that may in any way gain or lose financially from the publication of this manuscript, either now or in the future?
- Do you hold or are you currently applying for any patents relating to the content of the manuscript?
- Have you received reimbursements, fees, funding, or salary from an organization that holds or has applied for patents relating to the content of the manuscript?
- Do you have any other financial competing interests?
- Do you have any non-financial competing interests in relation to this paper?

If you can answer no to all of the above, write 'I declare that I have no competing interests' below. If your reply is yes to any, please give details below.

I declare that I have no competing interests

I agree to the open peer review policy of the journal. I understand that my name will be included on my report to the authors and, if the manuscript is accepted for publication, my named report including any attachments I upload will be posted on the website along with the authors' responses. I agree for my report to be made available under an Open Access Creative Commons CC-BY license (http://creativecommons.org/licenses/by/4.0/). I understand that any comments which I do not wish to be included in my named report can be included as confidential comments to the editors, which will not be published.

Choose an item.

To further support our reviewers, we have joined with Publons, where you can gain additional credit to further highlight your hard work (see: https://publons.com/journal/530/gigascience). On publication of this paper, your review will be automatically added to Publons, you can then choose whether or not to claim your Publons credit. I understand this statement.

Yes Choose an item.
