# Supplementary material for: ntsm: an alignment-free, ultra-low-coverage, sequencing technology agnostic, intraspecies sample comparison tool for sample swap detection
Source: Gigascience. 2024 Jun 4;13:giae024. doi: 10.1093/gigascience/giae024 (PMC11148594; doi:10.1093/gigascience/giae024)
Supplement: giae024_Reviewer_1_Report_Revision_1 — Jianxin Wang -- 2/28/2024 Reviewed [file giae024_reviewer_1_report_revision_1.pdf]

Reviewer Report

Title: ntsm: an alignment-free, ultra low coverage, sequencing technology agnostic, intraspecies sample comparison tool for sample swap detection

Version: Revision 1 **Date:** 2/28/2024

Reviewer name: Jianxin Wang

Reviewer Comments to Author:

In the revised manuscript, the authors addressed the issues I raised by conducting additional work. They presented more results to demonstrate that similarity score is a better metric for detecting sample swaps and the PCA-based screening method is effective. While Figure 7 shows the results for the Nanopore datasets, the error rates of these are not given. If possible, I hope the authors can provide the error rates of benchmark datesets. Here are some other issues.1. The format of references is messy. It need further examination and correction.2. The sentence "To observe the computational expense of this process we took subsamples of the 1000 Genomes VCF files and measured time and memory used to compute relatedness between the samples. " in section "Sample comparison process" may be incorrect. "compute relatedness" should be "detect sample swaps". I think ntsm_PCA cannot be used to compute relatedness.3. Running time and memory usage of ntsm for computing relatedness should be given.4. In Figure 3, the sentence "The Pearson correlation coefficient (R) is provided showing high correlation between k-mer based error rate estimate of the true error rate" is grammatically incorrect. "PCA-based screen approach" should be "PCA-based screening approach". "methods" in "To test our relatedness estimation methods" should be "method".

Level of Interest

Please indicate how interesting you found the manuscript: Choose an item.

Quality of Written English

Please indicate the quality of language in the manuscript: Choose an item.

Declaration of Competing Interests

Please complete a declaration of competing interests, considering the following questions:

- Have you in the past five years received reimbursements, fees, funding, or salary from an organisation that may in any way gain or lose financially from the publication of this manuscript, either now or in the future?
- Do you hold any stocks or shares in an organisation that may in any way gain or lose financially from the publication of this manuscript, either now or in the future?

- Do you hold or are you currently applying for any patents relating to the content of the manuscript?
- Have you received reimbursements, fees, funding, or salary from an organization that holds or has applied for patents relating to the content of the manuscript?
- Do you have any other financial competing interests?
- Do you have any non-financial competing interests in relation to this paper?

If you can answer no to all of the above, write 'I declare that I have no competing interests' below. If your reply is yes to any, please give details below.

'I declare that I have no competing interests.

I agree to the open peer review policy of the journal. I understand that my name will be included on my report to the authors and, if the manuscript is accepted for publication, my named report including any attachments I upload will be posted on the website along with the authors' responses. I agree for my report to be made available under an Open Access Creative Commons CC-BY license (http://creativecommons.org/licenses/by/4.0/). I understand that any comments which I do not wish to be included in my named report can be included as confidential comments to the editors, which will not be published.

Choose an item.

To further support our reviewers, we have joined with Publons, where you can gain additional credit to further highlight your hard work (see: https://publons.com/journal/530/gigascience). On publication of this paper, your review will be automatically added to Publons, you can then choose whether or not to claim your Publons credit. I understand this statement.

Yes Choose an item.
