# Supplementary material for: ntsm: an alignment-free, ultra-low-coverage, sequencing technology agnostic, intraspecies sample comparison tool for sample swap detection
Source: Gigascience. 2024 Jun 4;13:giae024. doi: 10.1093/gigascience/giae024 (PMC11148594; doi:10.1093/gigascience/giae024)
Supplement: giae024_Reviewer_2_Report_Original_Submission — Qian Zhou -- 12/28/2023 Reviewed [file giae024_reviewer_2_report_original_submission.pdf]

Reviewer Report

Title: ntsm: an alignment-free, ultra low coverage, sequencing technology agnostic, intraspecies sample comparison tool for sample swap detection

Version: Original Submission **Date:** 12/28/2023

Reviewer name: Qian Zhou

Reviewer Comments to Author:

In this paper, the authors have presented a tool, ntsm, which utilizes the k-mer distribution information directly from raw sequencing data for sample swap detection. The approach of bypassing the reference genome alignment step and saving computational resources is commendable. Utilizing k-mers for reference-free and de novo analysis of sequencing data is a valuable application. The authors have demonstrated the impressive performance of ntsm on low coverage data through experimental results presented in the manuscript, showcasing its strengths in terms of sensitivity, accuracy. However, while ntsm eliminates the need for reference genome alignment, it still relies on a pre-defined set of variant sites and pre-built PCA rotation matrices. This raises doubts about the true reference-free nature of ntsm and raises concerns about its generalizability to other species.Major comments:1.The concept of reference-free:I believe that ntsm's approach is not truly reference-free. In order to use ntsm, it requires the use of existing high-quality population SNP sites and kmers from the human reference genome. Additionally, the population PCA results are used to assist in pairwise comparisons between samples. Both of these information can only be obtained when a reference genome is available. A true reference-free tool would be applicable to species without a reference genome, such as SPLASH (Chaung et al., 2023, Cell). ntsm can be considered as an alignment-free or kmer-based tool.2.The reduction of computational costs:NTSM differs from Somalier in its computational workflow. To compare the computational costs or time, a holistic end-to-end comparison is necessary, rather than timing individual steps such as kmer counting and sample pairwise comparison separately. Conducting an end-to-end comparison for an analysis task allows users to have a comprehensive understanding of the tool's time and cost consumption. Furthermore, when comparing software, it is important to allocate computational resources fairly. For example, ntsm utilizes 16 threads in the 'Sample comparison process' stage, while for the 'k-mer counting (ntsm) vs. alignment (somalier)' stage, tools like bwa and minimap2, which can utilize multiple threads, were run using a single thread.3.Sensitivity and Specificity:More experimental details are needed. In the section 'Sensitivity and Specificity of Sample Swaps,' were the results obtained using the 39 HPRC samples? Did it include their Hi-C data?For Fig 6, did the results come from all sequencing datasets of the 39 samples, including Illumina and ONT? Since the results was obtained using full coverage, would the threshold change at lower coverage?For Fig 7, which demonstrates ntsm's results, was PCA information used as an auxiliary? Does the use of PCA information impact Sensitivity and Specificity?4.Regarding PCA-based method:The 39 HPRC samples used in the study are actually part of the 3,202 samples from the 1000 Genomes Project. Therefore, it is important to clarify whether the PCA matrix used in the study already includes information from these 39 samples. From a rigorous experimental design perspective, a precomputed PCA matrix should not

include information from the 39 samples. Otherwise, the effect of the PCA matrix on these 39 samples may be overestimated. It raises questions about whether the same results can be achieved on non-1000 Genomes Project samples.5.The applicability of the tool:In order to expand the applicability of ntsm to a wider range of species, two aspects need to be addressed:1). Provide detailed information on customizing the sites file. From the site files available in ntsm code repository on GitHub, the process of selecting variant sites seems to be more complex than what is described in the manuscript, involving more than just SNP variants.2). The sites and PCA files should be user-customizable inputs instead of being built-in. This limitation restricts the application of ntsm to other species.Minor comments:The manuscript appears to have been hastily written and requires further polish by the authors.1. In Figure 6, A and B seem to be labeled incorrectly.2. In Figure 9, the two subplots have different y-axes, one labeled "min" and the other labeled "s." Could you clarify what each subplot is illustrating?3. When mentioning HPRC for the first time, it would be helpful to provide the full name and explanation of the acronym. However, the full explanation appears in the next paragraph.4. "We then keep only purine to pyrimidine (A or T to G or C) variants, as final insurance against possible human error influencing this tool" It seems there may be a mistake or confusion in the sentence. The writer should indeed mention "A/G <-> C/T" instead of "A/T <-> G/C" to accurately describe purine to pyrimidine variants. The writer may have made an error in describing the nucleotide exchange, or it could be a typographical mistake.5. There is a typo in the formula for estimating sequencing error rate. (nm)Â·log(1-… …

Level of Interest

Please indicate how interesting you found the manuscript: Choose an item.

Quality of Written English

Please indicate the quality of language in the manuscript: Choose an item.

Declaration of Competing Interests

Please complete a declaration of competing interests, considering the following questions:

- Have you in the past five years received reimbursements, fees, funding, or salary from an organisation that may in any way gain or lose financially from the publication of this manuscript, either now or in the future?
- Do you hold any stocks or shares in an organisation that may in any way gain or lose financially from the publication of this manuscript, either now or in the future?
- Do you hold or are you currently applying for any patents relating to the content of the manuscript?
- Have you received reimbursements, fees, funding, or salary from an organization that holds or has applied for patents relating to the content of the manuscript?
- Do you have any other financial competing interests?
- Do you have any non-financial competing interests in relation to this paper?

If you can answer no to all of the above, write 'I declare that I have no competing interests' below. If your reply is yes to any, please give details below.

No competing interests.

I agree to the open peer review policy of the journal. I understand that my name will be included on my report to the authors and, if the manuscript is accepted for publication, my named report including any attachments I upload will be posted on the website along with the authors' responses. I agree for my report to be made available under an Open Access Creative Commons CC-BY license (http://creativecommons.org/licenses/by/4.0/). I understand that any comments which I do not wish to be included in my named report can be included as confidential comments to the editors, which will not be published.

Choose an item.

To further support our reviewers, we have joined with Publons, where you can gain additional credit to further highlight your hard work (see: https://publons.com/journal/530/gigascience). On publication of this paper, your review will be automatically added to Publons, you can then choose whether or not to claim your Publons credit. I understand this statement.

Yes Choose an item.
