# Supplementary material for: ntsm: an alignment-free, ultra-low-coverage, sequencing technology agnostic, intraspecies sample comparison tool for sample swap detection
Source: Gigascience. 2024 Jun 4;13:giae024. doi: 10.1093/gigascience/giae024 (PMC11148594; doi:10.1093/gigascience/giae024)
Supplement: giae024_Reviewer_2_Report_Revision_1 — Qian Zhou -- 2/19/2024 Reviewed [file giae024_reviewer_2_report_revision_1.pdf]

**Reviewer Report**

**Title: ntsm: an alignment-free, ultra low coverage, sequencing technology agnostic, intraspecies sample comparison tool for sample swap detection**

**Version: Revision 1** **Date:** 2/19/2024

**Reviewer name: Qian Zhou**

**Reviewer Comments to Author:**

I thank authors for taking time and giving detailed response to my commentsï¼Œwhich have resolved most of my major concerns in the previous round. I however still have a few comments related to the time comparisons.The author clearly divides the workflow of Somalier and ntsm into step 1 and step 2. This is reasonable. However, the comparisons lack a presentation of the total time consumption for step 1 and step 2. As a reader, what I see is that Figure 8 shows that in step 1, ntsm is much faster than Somalier, while Figure 9 shows that in step 2, ntsm is much slower than Somalier, especially when considering that Figure 9 uses log scale on the axes, indicating a significant difference between ntsm and Somalier in step 2. This does not allow the reader to quickly reach the conclusion stated in the abstract, "this tool (ntsm) is faster than alignment, it can save an immense degree of computational resources." I strongly recommend that the author use a table to present the time consumption for step 1, step 2, and the overall workflow for sample quantities such as 10, 100, 1000, 3000, etc.Additionally, are the results in Figure 8 based on a single sample or on all samples from Table S1? Can ntsm use multiple threads in the k-mer counting step? By using multiple threads in the k-mer counting step, it further reduces the time consumption of step 1 and enhances the practical applicability of ntsm in large cohort studies. After all, alignment is almost an unavoidable step in low-coverage whole-genome re-sequencing projects, while k-mer counting is not. Only when the k-mer counting step is fast enough, performing k-mer counting for sample swap detection will not add significant computational burden to the entire project.Please consider the following suggestions for the descriptions of results:1. "Somalier, utilizing a bitvector based comparison method is much more optimized for speed than our count based method, however we show that our PCA-based screening method is still competitive."This conclusion was derived when ntsm was using 16 threads. However, in the new Figure 9, the speed of the PCA-based screening method is significantly slower than Somalier, when both ntsm and Somalier are using a single thread.2. "Unlike Somalier, ntsm is capable of using more than one thread which can help close the gap in speed."The author points out that ntsm can alleviate the speed disadvantage in step 2 by using multiple threads. However, the author overlooks the fact that Somalier can also mitigate the speed disadvantage in step 1 by utilizing the multi-threading capabilities of tools like BWA and Minimap2. To ensure fairness in the description, it is suggested to remove this statement.Typos:1. In Response to Reviewers " we have corrected the formula to (n-n)Â·log(1-…", it should be "n-m".2. "Though most of the time reads will be used as input, Both Somalier and ntsm" , it should be "both".

**Level of Interest**

Please indicate how interesting you found the manuscript: Choose an item.

**Quality of Written English**

Please indicate the quality of language in the manuscript: Choose an item.

**Declaration of Competing Interests**

Please complete a declaration of competing interests, considering the following questions:

- Have you in the past five years received reimbursements, fees, funding, or salary from an organisation that may in any way gain or lose financially from the publication of this manuscript, either now or in the future?
- Do you hold any stocks or shares in an organisation that may in any way gain or lose financially from the publication of this manuscript, either now or in the future?
- Do you hold or are you currently applying for any patents relating to the content of the manuscript?
- Have you received reimbursements, fees, funding, or salary from an organization that holds or has applied for patents relating to the content of the manuscript?
- Do you have any other financial competing interests?
- Do you have any non-financial competing interests in relation to this paper?

If you can answer no to all of the above, write 'I declare that I have no competing interests' below. If your reply is yes to any, please give details below.

I declare that I have no competing interests.

I agree to the open peer review policy of the journal. I understand that my name will be included on my report to the authors and, if the manuscript is accepted for publication, my named report including any attachments I upload will be posted on the website along with the authors' responses. I agree for my report to be made available under an Open Access Creative Commons CC-BY license (http://creativecommons.org/licenses/by/4.0/). I understand that any comments which I do not wish to be included in my named report can be included as confidential comments to the editors, which will not be published.

Choose an item.

To further support our reviewers, we have joined with Publons, where you can gain additional credit to further highlight your hard work (see: https://publons.com/journal/530/gigascience). On publication of this paper, your review will be automatically added to Publons, you can then choose whether or not to claim your Publons credit. I understand this statement.

Yes Choose an item.
